# From Batch to Pilot: Scaling Up Arsenic Removal with an Fe-Mn-Based Nanocomposite

**DOI:** 10.3390/nano15141104

**Published:** 2025-07-16

**Authors:** Jasmina Nikić, Jovana Jokić Govedarica, Malcolm Watson, Đorđe Pejin, Aleksandra Tubić, Jasmina Agbaba

**Affiliations:** Department of Chemistry, Biochemistry and Environmental Protection, Faculty of Sciences, University of Novi Sad, Trg Dositeja Obradovića 3, 21000 Novi Sad, Serbia; jasmina.nikic@dh.uns.ac.rs (J.N.); jovanaj@dh.uns.ac.rs (J.J.G.); djordje.pejin@dh.uns.ac.rs (Đ.P.); aleksandra.tubic@dh.uns.ac.rs (A.T.); jasmina.agbaba@dh.uns.ac.rs (J.A.)

**Keywords:** arsenic, drinking water, Fe-Mn nanocomposite, pilot scale, breakthrough models, adsorption capacity, column tests

## Abstract

Arsenic contamination in groundwater is a significant public health concern, with As(III) posing a greater and more challenging risk than As(V) due to its higher toxicity, mobility, and weaker adsorption affinity. Fe-Mn-based adsorbents offer a promising solution, simultaneously oxidizing As(III) to As(V), enhancing its adsorption. This study evaluates an Fe-Mn nanocomposite across typical batch (20 mg of adsorbent), fixed-bed column (28 g), and pilot-scale (2.5 kg) studies, bridging the gap between laboratory and real-world applications. Batch experiments yielded maximum adsorption capacities of 6.25 mg/g and 4.71 mg/g in a synthetic matrix and real groundwater, respectively, demonstrating the impact of the water matrix on adsorption. Operational constraints and competing anions led to a lower capacity in the pilot (0.551 mg/g). Good agreement was observed between the breakthrough curves in the pilot (breakthrough at 475 bed volumes) and the fixed-bed column studies (365–587 bed volumes) under similar empty bed contact times (EBCTs). The Thomas, Adams–Bohart, and Yoon–Nelson models demonstrated that lower flow rates and extended EBCTs significantly enhance arsenic removal efficiency, prolonging the operational lifespan. Our findings demonstrate the necessity of continuous-flow experiments using real contaminated water sources and the importance of optimizing flow conditions, EBCTs, and pre-treatment in order to successfully scale up Fe-Mn-based adsorbents for sustainable arsenic removal.

## 1. Introduction

Arsenic contamination in groundwater poses significant challenges to public health and environmental safety, particularly in regions where geochemical and anthropogenic activities contribute to elevated arsenic levels [1,2,3]. Chronic exposure to arsenic, even at low concentrations, has been linked to severe health risks, including cancer, cardiovascular disease, and neurological disorders [4,5]. Consequently, the development of efficient, scalable, and sustainable technologies for arsenic removal is a critical area of research.

Different methods are available for arsenic removal from water, including coagulation–flocculation, ion exchange, membrane filtration, electrocoagulation, phytoremediation, adsorption, and others [6,7,8]. While each of these technologies has demonstrated high removal efficiencies under specific conditions, they also present notable limitations. Coagulation requires chemical addition and generates sludge. Ion exchange resins require frequent regeneration, are often selective for only one arsenic species, and their performance can be compromised by competing anions due to an unfavorable selectivity order. Additionally, they are expensive and may require pH adjustments that introduce corrosion risks. Membrane technologies, though effective, are associated with high operational costs and significant water rejection. Electrocoagulation processes produce sludge that requires further handling and disposal and are highly influenced by the type and dose of reagents, solution pH, and competing ions. Phytoremediation, while eco-friendly and socially acceptable, is a slow process and highly dependent on climate, plant species, and site-specific conditions [6,7,8].

Given these constraints, adsorption has emerged as a highly effective and versatile approach for arsenic remediation due to its simplicity, cost-effectiveness, and adaptability to various operational conditions in both centralized and decentralized systems [6,7,8]. It enables the efficient removal of both As(III) and As(V), especially when using advanced materials such as Fe–Mn-based adsorbents, which exhibit dual oxidative–adsorptive functionality. These materials have garnered significant attention for their ability to oxidize As(III) and subsequently adsorb As(V), leveraging the high affinity of iron and manganese oxides for arsenic species [9,10,11,12,13]. This combined mechanism makes Fe–Mn-based adsorbents highly efficient in treating a wide range of arsenic contamination scenarios [14]. While several Fe–Mn-based adsorbents have been extensively studied, and even patented, for arsenic removal, our work contributes to this field by integrating batch, column, and pilot-scale experiments using real arsenic-contaminated groundwater. This approach provides practical insights into adsorbent performance under realistic conditions, an aspect that remains insufficiently addressed in the existing literature.

The majority of studies on Fe–Mn-based adsorbents primarily focus on batch adsorption experiments, often neglecting evaluation under continuous operational conditions. This is largely due to the complexity of establishing dynamic flow systems compared to batch setups. Although batch studies are valuable for determining the equilibrium and kinetic parameters, they do not replicate the complexities of real-world water treatment. Furthermore, the initial arsenic concentrations used in batch experiments are often significantly higher than those typically found in natural groundwater. This disparity is critical, as there is generally a strong correlation between the initial contaminant concentration and the maximum adsorption capacity (qmax) obtained in batch studies, regardless of the adsorbent used (see Supplementary Material Figure S1). As shown in Table 1, batch adsorption studies using Fe–Mn-based adsorbents typically employ initial arsenic concentrations ranging from 0.01 to 300 mg/L, depending on the adsorbent type and study objective. These concentrations are often substantially higher than those commonly found in naturally contaminated groundwater. According to global assessments, arsenic concentrations in groundwater often exceed the WHO guideline value of 10 µg/L, with typical levels ranging from 50 to 500 µg/L in affected regions. In extreme cases, concentrations as high as 4600 µg/L have been reported in Bangladesh, 3880 µg/L in India, and up to 14,969 µg/L in parts of Latin America [1]. In Serbia, groundwater in the Vojvodina region has shown arsenic levels reaching up to 350 µg/L [15]. This quantitative disparity underscores a key limitation of batch experiments—they may not adequately predict adsorbent performance under environmentally realistic conditions. Although Fe–Mn-based adsorbents exhibit high adsorption capacities in batch experiments (Table 1), their practical performance in real-world applications may be hindered by factors such as flow dynamics, the presence of competing ions, and long-term operational stability [16,17]. To overcome these limitations and ensure the scalability of new adsorbent technologies, performance testing under continuous-flow conditions is essential. Column and pilot-scale experiments enable the assessment of adsorbents under conditions that better simulate real groundwater treatment scenarios [18,19,20].

Continuous-flow studies provide critical insights into the dynamic behavior of adsorbents, allowing researchers to examine performance under practical operational parameters such as flow rate, breakthrough volume, bed height, empty bed contact time (EBCT), pH, and ionic competition [31]. These investigations also help evaluate the adsorbent regeneration potential, structural stability, and operational robustness. Pilot-scale studies further advance this understanding by simulating near-industrial conditions, enabling long-term performance evaluation and the identification of potential bottlenecks. By integrating continuous-flow column and pilot-scale testing, researchers can bridge the gap between laboratory-scale findings and real-world applications, thus accelerating the development of reliable and scalable arsenic removal technologies [32].

To the best of our knowledge, only a limited number of studies have evaluated Fe–Mn-based adsorbents for arsenic removal, particularly As(III), under continuous-flow conditions [11,24,27,29,33,34]. Even fewer have tested their performance using real groundwater matrices [16,35], highlighting the need for further research in continuous treatment systems to provide a more reliable assessment of the suitability of Fe–Mn-based adsorbents for scalable and sustainable arsenic removal solutions. This study aims to bridge the gap between theoretical and practical adsorption capacities by integrating batch, column, and pilot-scale experiments to assess an Fe–Mn nanocomposite under realistic operational scenarios. Comparative analyses across different experimental scales were conducted to evaluate adsorption efficiency and operational feasibility in real groundwater treatment conditions. Batch adsorption experiments were modeled using the Freundlich and Langmuir isotherms to describe equilibrium behavior and adsorption capacity, providing initial insights into arsenic affinity under controlled conditions. To assess adsorbent performance under dynamic conditions, continuous-flow column and pilot-scale experiments were conducted using real arsenic-contaminated groundwater. Breakthrough curves were modeled using the Thomas, Adams–Bohart, and Yoon–Nelson models, enabling the prediction of adsorption performance, breakthrough time, and bed saturation under real operational conditions. These models provided a deeper understanding of the influence of flow rate, empty bed contact time (EBCT), and competitive adsorption effects from naturally occurring anions such as phosphate. Overall, these findings highlight the necessity of continuous-flow studies in adsorption research to ensure a more reliable evaluation of Fe–Mn-based adsorbents for large-scale water treatment applications.

## 2. Materials and Methods

### 2.1. Materials

The Fe–Mn nanocomposite adsorbent used in this study was developed for arsenic removal from groundwater. It is designed to oxidize As(III) and adsorb As(V), making it highly effective for water treatment applications. The material consists of iron and manganese oxides incorporated within a polymeric matrix, providing both mechanical stability and enhanced adsorption capacity. This combination allows for efficient contaminant removal while ensuring material durability and reusability. More exact details regarding the scaled-up synthesis process and material characterization cannot be disclosed, as they are subject to an intellectual property agreement. However, the adsorbent was tested for its performance in batch, column, and pilot-scale experiments to assess its arsenic removal efficiency under various conditions. The particle size of the FMBO nanocomposite was 0.3–1.2 mm, and the packing density of the material used in the fixed-bed experiments was 0.52 g/cm^3^, determined based on the dry mass and packed bed volume.

### 2.2. FMBO Nanocomposite Characterization

The textural characteristics of the adsorbents, including the specific surface area, pore size, and pore volume, were determined using an Autosorb iQ Surface Area Analyzer (Quantachrome Instruments, Boynton Beach, FLUSA). Specific surface areas were calculated using the multi-point Brunauer–Emmett–Teller (BET) approach, while mesopore and micropore volumes were estimated from the desorption branch of the isotherms using the Barrett–Joyner–Halenda (BJH) method and the t-plot technique, respectively.

The morphology and surface elemental composition of the materials were analyzed through scanning electron microscopy (SEM) with a TM3030 instrument (Hitachi High-Technologies, Tokyo, Japan), equipped with an energy dispersive X-ray spectroscopy (EDS) system (Quantax 70, Bruker Nano GmbH, Berlin, Germany).

Functional groups present on the adsorbent surfaces were identified by Fourier-transform infrared (FTIR) spectroscopy using a Nicolet iS20 spectrometer (Thermo Fisher Scientific, Waltham, MA, USA), operated in diffuse reflectance mode at a resolution of 4 cm^−1^.

The point of zero charge (pHpzc) was determined following the pH drift method in 0.1 M NaNO_3_ electrolyte solutions, with initial pH values ranging from 2 to 10, as previously described by Nikić et al. [16].

### 2.3. Investigated Water Matrices

The batch and column experiments were conducted using both arsenic-contaminated groundwater collected from a well in the northern part of Serbia (Autonomous Province of Vojvodina) and tap water spiked with As(III), whereas the pilot study exclusively used arsenic-contaminated groundwater. As explained below, the groundwater was also subject to aeration and sand filtration to yield a third water matrix, aerated groundwater. The characteristics of the investigated water matrices are summarized in Table 2.

The groundwater sample exhibited a pH of 7.73 ± 0.05, indicating a slightly alkaline nature. The conductivity value of 514 ± 43 µS/cm suggests a moderate level of dissolved ions, likely originating from both geogenic sources and possible anthropogenic inputs. The relatively low turbidity (1.55 ± 0.29 NTU) indicates minimal suspended solids, implying that the groundwater is primarily affected by dissolved contaminants rather than particulate matter.

Arsenic was detected at 115 ± 6.40 µg/L, significantly exceeding the WHO drinking water limit of 10 µg/L. This elevated concentration is attributed to geogenic sources, particularly the dissolution of arsenic-bearing minerals within the aquifer. Arsenic speciation analysis confirmed that As(III) is the dominant species (>90%), which aligns with the mildly reducing conditions of the water. Under these conditions, arsenic remains more mobile and difficult to remove compared to As(V), which is typically present in oxidizing environments and exhibits a stronger affinity for adsorbent surfaces. This high arsenic concentration is mostly related to the geological characteristics of the region, which is known for elevated arsenic levels in sedimentary minerals, with concentrations reported up to 185 mg/kg [36]. Arsenic is naturally mobilized through desorption, dissolution, and reductive processes, especially under anoxic conditions in deeper aquifers. Besides geogenic sources, agricultural activities and sewage have been identified as primary contributors to arsenic pollution in parts of Vojvodina [37]. The presence of iron (0.395 ± 0.03 mg/L) and manganese (0.054 ± 0.005 mg/L) supports the interpretation of a reducing environment within the aquifer, as both elements are key indicators of redox processes that influence arsenic mobility. Under reducing conditions, Fe(III) and Mn(IV) are converted to Fe(II) and Mn(II), which can lead to the desorption and release of arsenic into groundwater. The coexistence of elevated iron and manganese concentrations together with As(III) suggests that the reductive dissolution of Fe/Mn oxyhydroxides is a likely driving mechanism for arsenic mobilization. These hydrogeochemical conditions promote the release of arsenic from mineral surfaces, such that Fe-bearing and Mn-bearing minerals are likely the primary sources of the dissolved arsenic in this groundwater [37]. The total organic carbon (TOC) concentration of 1.38 ± 0.10 mg/L indicates a moderate level of organic matter, which can influence arsenic mobility. Organic matter may enhance arsenic solubility by forming organo-arsenic complexes or facilitate its removal through interactions with Fe/Mn oxides [38]. The nitrate concentration (1.57 ± 0.14 mg N/L) is well below the WHO guideline of 50 mg/L, indicating minimal agricultural or industrial contamination. However, ammonium (0.44 ± 0.09 mg N/L) and orthophosphate (0.323 ± 0.11 mg PO4/L) were detected at measurable levels, suggesting microbial activity or anthropogenic inputs. Phosphate is a known competitor with arsenic for adsorption sites, which may reduce the efficiency of adsorbent-based arsenic removal processes [9,10,11,12,14,16,17,28].

In order to avoid clogging of the adsorbent surface with ferric hydroxide floccs, aerated groundwater was also investigated. The original groundwater was aerated for 1 h and passed through a sand filter column to remove the resulting ferric hydroxide floccs. After filtration, 98.6% of the iron was removed. During this process, 21.7% of the arsenic was also removed by co-precipitation. A significant 77% reduction in the phosphate concentration in the aerated water was also observed.

### 2.4. Batch Adsorption Experiment

Batch adsorption experiments were conducted to evaluate the adsorption capacity of the Fe–Mn nanocomposite under equilibrium conditions. Sorption isotherms were performed by spiking two different water matrices: groundwater naturally contaminated with arsenic and tap water. For each experiment, 20 mg of the sorbent was added to 40 mL glass vials containing 20 mL of the respective water matrix, with As(III) concentrations spiked to range from 0.1 mg/L to 10 mg/L. The samples were placed on a shaker at 180 rpm for 24 h to ensure equilibration. After the equilibration period, the supernatant was decanted and preserved with concentrated HNO_3_ before analysis. The concentrations of arsenic, iron, and manganese were determined by ICP/MS. The adsorption capacity at equilibrium was calculated based on the difference between the initial and final arsenic concentrations. All experiments were conducted in triplicate, with error bars representing standard deviations. To account for potential analytical errors and losses, control samples (without sorbent) were prepared under identical conditions for each water matrix. The arsenic concentrations in these control samples were used as reference initial concentrations for each experimental series.

The data obtained from these studies were modeled using the Freundlich and Langmuir isotherm models (Table 3) to determine the adsorption capacity, adsorption mechanism, and equilibrium parameters, which are critical for designing large-scale treatment systems.

To evaluate the reusability of the FMBO nanocomposite, three consecutive adsorption–desorption cycles were conducted under batch conditions. After the initial adsorption tests using As(III) solutions (initial concentration: 0.2 mg/L; pH 7.0; contact time: 24 h), the exhausted sorbent was regenerated using different regenerant solutions: 0.1 M NaOH, 0.5 M NaOH, and 1 M NaOH and a mixed solution containing 0.5 M NaOH, 0.1 M NaCl, and 0.01 M NaOCl for 2 h. The alkaline solutions (NaOH and NaCl) facilitated the desorption of arsenic species, while NaOCl served to oxidize Mn(II) back to Mn(IV), potentially restoring the redox capacity of the sorbent [24]. Following the desorption step, the supernatant was collected for arsenic concentration analysis, and the sorbent was thoroughly rinsed with distilled water until the effluent pH stabilized at 7.0. The regenerated adsorbent was then directly reused in the subsequent adsorption cycle under the same experimental conditions.

### 2.5. Column Adsorption Experiment

The continuous fixed-bed adsorption studies were conducted in glass columns with an internal diameter of 2 cm. A 0.5 cm thick layer of glass wool was placed at the bottom of the column to prevent adsorbent loss. The Fe–Mn nanocomposite was packed into the column in a predetermined quantity. Tap water spiked with As(III) to an initial concentration of 200 μg/L or real groundwater contaminated with arsenic (with and without pre-treatment via aeration) was pumped in a downflow mode at a constant flow rate using a peristaltic pump. To evaluate the performance of the Fe–Mn nanocomposite in a fixed-bed adsorption column, a series of experiments was conducted under various operational conditions, as detailed in Table S1 of the Supplementary Information. The study included four different column setups, treating either tap water spiked with As(III), untreated groundwater, or aerated groundwater.

Column I and Column II (60 cm length, 2 cm internal diameter) were used for the tap water spiked with As(III), both containing 131 mL of filter media with a bed depth of 41.7 cm. They were operated at different filtration and flow rates. Column I had a filtration rate of 1 m/h and an empty bed contact time (EBCT) of 25 min, whereas Column II had a higher filtration rate of 5 m/h and an EBCT of 5 min, allowing a comparison of adsorption efficiency under different flow conditions. Column III and Column IV (30 cm length, 2 cm internal diameter) were tested using natural groundwater and aerated groundwater, respectively. Both contained 62.8 mL of filter media with a bed depth of 20 cm, filtration rates of 1 m/h, and an EBCT of 12 min. The comparison between these two columns aimed to assess the influence of aeration on arsenic removal efficiency, as aeration can affect the oxidation state of arsenic and enhance adsorption performance. In this study, aeration was performed immediately prior to column loading using a simple aeration device, followed by sand filtration to simulate typical field conditions, where dissolved iron and manganese are oxidized and the resulting precipitates are removed through filtration. After this pre-treatment step, the aerated groundwater was introduced into the column packed with the FMBO nanocomposite.

The adsorption data collected from these experiments were further analyzed using mathematical models, including the Thomas, Adams–Bohart, and Yoon–Nelson models, to characterize adsorption behavior and predict breakthrough curves under continuous-flow conditions. Both the linear and nonlinear forms of these models are summarized in Table 4. In this study, adsorption data were fitted using the nonlinear forms of these models.

### 2.6. Pilot Experiment

Following the batch and column experiments, a pilot-scale study was conducted using a continuous-flow filtration unit to assess the efficiency of the Fe–Mn polymer nanocomposite for arsenic removal under real-world conditions. The system was installed on site at a waterworks with arsenic-contaminated groundwater. The filtration unit consisted of a pressure vessel column (20.3 cm in diameter, 43 cm in height) with a total volume of 10 L, filled with an Fe–Mn polymer nanocomposite adsorbent. The unit was fed arsenic-contaminated groundwater by a peripheral pump and included flowmeters, pressure gauges, sampling taps for water quality monitoring, and a backwash system utilizing treated water. The unit was run continuously with groundwater containing elevated As(III) levels, allowing for performance assessment under varying operational conditions (Table 5). Arsenic removal efficiency was determined by comparing influent and effluent concentrations using ICP-MS analysis. The effects of flow rate, EBCTs, and bed depth on the treatment performance were examined through modeling using the Thomas, Adams–Bohart, and Yoon–Nelson models (Table 4), while breakthrough curves were generated to assess media longevity and optimize replacement cycles.

### 2.7. Analytical Methods

The pH of the samples was measured using a FiveEasy Plus pH meter FP20-Std-Kit (Mettler-Toledo GmbH, Greifensee, Switzerland). The electrical conductivity of the groundwater and tap water was determined using a WTW Cond 3110 conductometer (Xylem Analytics Germany, Weilheim, Germany), following the SRPS EN 27888:2009 (Institute for Standardization of Serbia, Belgrade, Serbia, 2009) method [39]. Turbidity was measured using a Thermo Scientific Eutech TN-100 turbidimeter (Thermo Fisher Scientific, Waltham, MA, USA). The total organic carbon (TOC) content in the water was determined using the Elementar Vario TOC Select instrument (Langenselbold, Germany), according to the SRPS ISO 8245:2007 (Institute for Standardization of Serbia, Belgrade, Serbia, 2007) method [40]. The orthophosphate content was determined spectrophotometrically with ammonium molybdate, following the SRPS EN ISO 6878:2008 (Institute for Standardization of Serbia, Belgrade, Serbia, 2008) method [41]. The nitrate content was analyzed spectrophotometrically with sulfosalicylic acid, according to the SRPS ISO 7890-3:1998 (Institute for Standardization of Serbia, Belgrade, Serbia, 1998) method [42]. The chloride content was determined titrimetrically using silver nitrate with a chromate indicator (Mohr’s method), following the SRPS ISO 9297/1:2007 (Institute for Standardization of Serbia, Belgrade, Serbia, 2007) method [43]. The ammonium concentration in the water was determined spectrophotometrically using the SRPS H.Z1.184:1974 (Institute for Standardization of Serbia, Belgrade, Serbia, 1974) method [44].

The concentrations of arsenic, iron, and manganese in the water samples were determined using inductively coupled plasma mass spectrometry (ICP-MS) with a mass detector (Agilent Technologies 7700 Series ICP-MS) (Tokyo, Japan), following US EPA method 6020B (Washington, DC, USA) [45]. The method detection limit (MDL) for arsenic, iron, and manganese was 0.001 mg/L. Arsenic speciation in the water samples (both raw and treated) was performed using solid-phase extraction (SPE) at pH 5.6 with a Visiprep SPE Vacuum Manifold system (Sigma-Aldrich, St. Louis, MO, USA). For the separation of arsenic species, a strong anion-exchange resin (LC-SAX, Supelco) (Bellefonte, PA, USA) was used. The SPE column was conditioned with 5 mL of methanol twice, followed by 5 mL of deionized water three times. The water sample was then passed through the LC-SAX resin, during which As(III) was retained. After that, the resin was eluted with 1 M HNO_3_ to recover As(V).

## 3. Results and Discussion

### 3.1. FMBO Nanocomposite Chracterization

Characterization of the FMBO nanocomposite included determination of the textural properties (specific surface area, pore volume, and pore size), surface morphology, and chemistry (SEM/EDS, XRD, and FTIR), as well as the point of zero charge (pHpzc).

The specific surface area of the FMBO nanocomposite was 39.8 m^2^/g. The t-plot analysis did not detect micropores, indicating the dominant presence of larger pores within the material’s structure. The mesopore volume and average pore diameter could not be determined using standard methods due to the absence of a well-defined desorption branch in the isotherm, suggesting a predominantly macroporous structure or heterogeneous porosity not fitting conventional micro/mesopore definitions. Although the nanocomposite material shows limited porosity in terms of classical micropore/mesopore distribution, it retains a significant adsorption capacity and exhibits improved stability, mechanical strength, and suitability for use in continuous systems, such as water treatment columns, in comparison to FMBO nanoparticles.

To assess the morphological characteristics of the FMBO nanocomposite, SEM/EDS analysis was performed (Figure S2). The SEM micrographs revealed that the surface was coated with a layer of agglomerated Fe–Mn binary oxide particles of varying sizes. These observations confirm the successful dispersion of binary oxide particles onto the polymeric support and the development of a new material with improved properties, including a higher number of active adsorption sites [13,16]. EDS analysis confirmed the presence of iron (Fe) and manganese (Mn) on the surface of the nanocomposite, with mass fractions of 14.34% and 4.96%, respectively. This confirms the successful deposition of Fe–Mn binary oxide onto the polymer matrix. The high content of carbon (70.48%) and oxygen (8.11%) corresponds to the organic polymeric matrix and the presence of oxide functional groups. Other detected elements in trace amounts are attributed to residual synthesis reagents or minor impurities.

The crystallographic structure of the FMBO nanocomposite was determined using X-ray diffraction (XRD) (Rigaku MiniFlex 600 diffractometer, (Tokyo, Japan)), and the results are presented in Figure S3. The XRD pattern revealed broad, low-intensity peaks characteristic of amorphous materials. Sharp, well-defined crystalline peaks were not observed, indicating the absence of crystalline phases. This phase structure was expected and is a direct consequence of the applied synthesis method, which involved co-precipitation of iron and manganese oxides onto a polymeric support. Moreover, the broad maxima and reduced peak intensities further confirm that the iron and manganese oxides forming the active surface layer on the polymer support are present in an amorphous form. These results are in line with literature reports describing similar nanocomposites [13,16,28].

As shown in Figure S4, the FTIR spectra exhibited absorption bands in the range of 2707–2956 cm^−1^, which are attributed to the stretching vibrations of C–H bonds from –CH_3_ and –CH_2_ groups, as well as other functional groups characteristic of the polymer matrix structure. The bands observed at 545 and 425 cm^−1^ correspond to the vibrational modes of Fe–O and/or Mn–O bonds, confirming the presence of metal oxide components within the FMBO nanocomposite structure [13,23,26].

The point of zero charge (pHpzc) is a key parameter in understanding arsenic adsorption mechanisms, as it directly influences the surface charge of the adsorbent and the predominant arsenic species in water. The pHpzc of the FMBO nanocomposite was found to be 8.80, indicating that the adsorbent surface is positively charged at pH < 8.80 and negatively charged at pH > 8.80. Given that the pH of the investigated groundwater was 7.73 ± 0.05 (Table 1), the surface of the FMBO nanocomposite is positively charged. This favors electrostatic attraction with negatively charged As(V) species, such as H_2_AsO_4_^−^ and HAsO_4_^2−^, thereby facilitating their adsorption. In contrast, As(III) predominantly exists in its neutral molecular form (H_3_AsO_3_) at this pH and is not subject to electrostatic interactions. Consequently, the removal of As(III) occurs via a two-step process. In the first step, As(III) is oxidized to As(V) by manganese oxides incorporated within the nanocomposite matrix. In the second step, the newly formed As(V) species are efficiently adsorbed onto the positively charged surface of the FMBO nanocomposite. This combined oxidation–adsorption mechanism enables the effective removal of both arsenic species and has been consistently observed in studies using Fe–Mn-based adsorbents [11,13,14,16,21,22,23,24,25,26]. Overall, the arsenic removal process involves synergistic contributions from redox reactions, electrostatic interactions, and surface complexation, depending on the oxidation state of arsenic and the prevailing pH conditions.

### 3.2. Batch Adsorption Study

**Figure 1:**
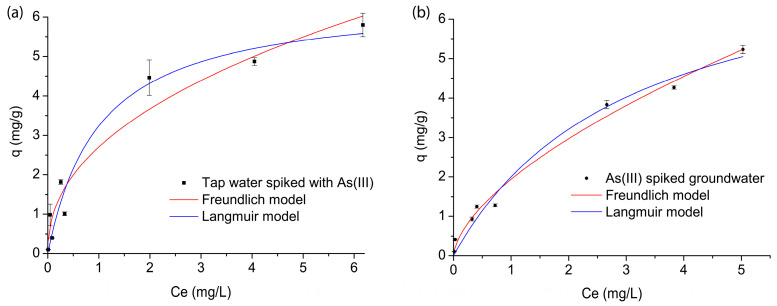
Freundlich and Langmuir adsorption isotherms of arsenic adsorption on an FMBO nanocomposite. (**a**) Tap water spiked with As(III), and (**b**) spiked groundwater (C0 = 0.1–10 mg/L, pH = 7.02 ± 0.2).

The adsorption affinity of the FMBO nanocomposite for arsenic was evaluated through batch experiments conducted under equilibrium conditions using two different water matrices: tap water spiked with As(III) and arsenic-contaminated groundwater (Figure 1). The experimental data obtained were further analyzed using the two most commonly applied isotherm models, Freundlich and Langmuir, to determine key adsorption parameters such as adsorption capacity and intensity. The corresponding parameters of the applied models are summarized in Table 6.

Based on the coefficient of determination (R^2^) values, both models showed a strong correlation with the experimental data (R^2^ > 0.93). However, the Langmuir model showed better agreement with the experimental data (R^2^ > 0.94). The Freundlich model, which describes multilayer adsorption on a heterogeneous surface, indicated that adsorption was more favorable in the spiked tap water (n = 0.640) than in the real groundwater (n = 0.420), as expected. The higher value of the Freundlich constant (K_F_ = 2.70 mg/g (mg/L)^0.64^) in the spiked water compared to 1.79 mg/g (mg/L)^0.42^ in the groundwater suggests that arsenic adsorption was more efficient in the cleaner tap water matrix. In the real groundwater, the presence of competing anions, primarily phosphate, reduced the number of adsorption sites available for arsenic. The Langmuir model, which assumes monolayer adsorption on a homogeneous surface, showed a higher theoretical maximum adsorption capacity (q_max_) in the spiked tap water (6.25 mg/g) than in the groundwater (4.63 mg/g), which is in agreement with Freundlich K_F_ values. Additionally, the Langmuir binding affinity constant (K_L_) was significantly higher in the spiked tap water (0.992 L/mg) than in the groundwater (0.343 L/mg), indicating stronger arsenic binding in controlled conditions. The lower K_L_ value in the groundwater confirms that arsenic adsorption is less efficient due to competition with naturally occurring ions.

### 3.3. Column Adsorption Study

**Figure 2:**
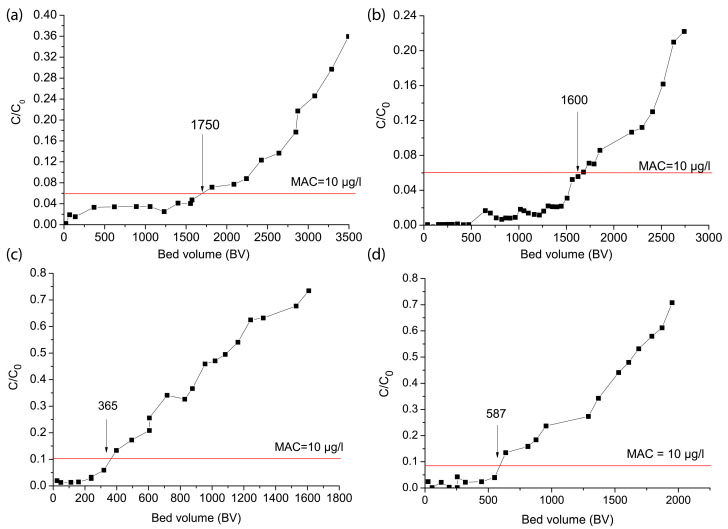
Breakthrough curves for arsenic removal using the Fe-Mn nanocomposite. (**a**,**b**) Columns I and II: tap water spiked with As(III), (**c**) aerated water in Column III, and (**d**) groundwater in Column IV.

After the batch adsorption experiments, we further investigated the performance of the Fe–Mn nanocomposite for arsenic removal through column experiments, ensuring a comprehensive evaluation of its effectiveness under continuous operational conditions. Arsenic removal by the Fe–Mn nanocomposite was evaluated under four distinct conditions. In Columns I and II, tap water spiked with As(III) was passed through the column at different flow rates (5.2 mL/min and 26.2 mL/min), corresponding to empty bed contact times (EBCTs) of 25 min and 5 min, respectively. In Columns III and IV, real groundwater containing arsenic—both with and without aeration—was used as the feed solution, with both columns operating at a constant flow rate of 5.2 mL/min (Table S1, Supplementary Material). The breakthrough curves illustrating arsenic removal under these different conditions are presented in Figure 2. Breakthrough curves as a standard method illustrate arsenic removal in the continuous-flow column experiments, showing effluent concentration changes over time, whereby the breakthrough point was defined as the stage at which the arsenic concentration in the treated water exceeded the regulatory limit of 10 μg/L. In Figure 2, the breakthrough curves are depicted as C/C_0_ vs. the bed volume. Bed volume is the volume of water treated expressed as multiples of the adsorbent volume. The initial arsenic concentration in the tap water feed for Columns I and II was 172 ± 20 μg/L, with the breakthrough point for total arsenic occurring at 10 μg/L, corresponding to a C/C_0_ ratio of 0.058. The initial arsenic concentrations in the groundwater and aerated groundwater were 120.3 ± 18.2 μg/L (C/C_0_ ratio of 0.08) and 90 ± 12 μg/L (C/C_0_ ratio of 0.11), respectively.

Based on the obtained results, the bed volumes (BVs) treated before breakthrough varied significantly based on the flow rate, empty bed contact time (EBCT), and water matrix composition. In Column I and Column II, where tap water spiked with As(III) was treated, arsenic breakthrough occurred after 1750 BV and 1600 BV, respectively (Figure 2a,b). A longer empty bed contact time (EBCT) of 25 min in Column I, compared to the shorter EBCT of 5 min in Column II, facilitated more effective arsenic removal. The extended contact duration allowed for improved interaction between the Fe–Mn nanocomposite and arsenic in solution, enhancing the adsorption process [23]. Similarly, Asif et al. [46] observed that increasing the flow rate led to a faster breakthrough, suggesting that at lower flow rates, the adsorbent takes more time to reach saturation. This phenomenon occurs because higher flow rates reduce the residence time of the adsorbate in the column, limiting its ability to interact with the adsorbent. In contrast, at lower flow rates, metal ions have more time to diffuse into the pores of the adsorbent, allowing for more effective adsorption via intra-particle diffusion. Conversely, for the higher flow rate, the arsenic solution will leave the column bed before the equilibrium can be reached.

In contrast, the real groundwater columns (III and IV) achieved significantly lower BVs before breakthrough. In the aerated groundwater (Column III), breakthrough occurred at 365 BV, while in the non-aerated groundwater (Column IV), breakthrough occurred at 587 BV (Figure 2c,d). Interestingly, aeration did not enhance arsenic removal efficiency as initially expected. Instead, it negatively impacted performance, despite iron concentrations decreasing significantly after aeration (from 395 μg/L to 5.36 μg/L). This reduction in iron likely diminished the co-precipitation effect, which otherwise could have enhanced arsenic removal. In contrast, non-aerated groundwater (Column 4) retained a higher iron concentration (395 μg/L), facilitating arsenic removal via co-precipitation and surface complexation with the Fe–Mn nanocomposites. Although Column IV exhibited slightly improved breakthrough performance (587 BV), overall arsenic removal remained much lower than in the tap water columns. This difference can be attributed to the competing anions present in the groundwater, primarily phosphate (0.401 mg PO_4_/L in groundwater), which likely inhibited arsenic adsorption by competing for active sites on the surface of the Fe–Mn nanocomposite [9,10,11,12,14,16,17,28]. Residual concentrations of Fe and Mn in the water after treatment with the Fe–Mn nanocomposite were below the maximum allowable limits of 0.3 mg/L and 0.05 mg/L, respectively, confirming the stability of the Fe–Mn nanocomposite adsorbent, as shown in Table S2 of the Supplementary Information.

The performance of the Fe–Mn nanocomposite in fixed-bed column studies was further compared with other Fe–Mn-based adsorbents used in continuous-flow systems to evaluate its adsorption efficiency for As(III) and breakthrough volume (BV) under realistic conditions (Table 7). The Fe–Mn nanocomposite exhibited a breakthrough volume of 1700 BV in the spiked tap water and 587 BV in the non-aerated groundwater, demonstrating competitive performance compared to other Fe–Mn adsorbents, particularly in real groundwater conditions, where adsorption efficiency is often hindered by competing ions.

Several Fe–Mn-based adsorbents tested in synthetic or simulated waters have reported higher breakthrough volumes. For instance, chitosan-coated Fe–Mn binary oxide achieved 3200 BV for As(III) under controlled conditions [29], while macroporous anion exchanger-supported Fe–Mn binary oxide attained 2300 BV in a simulated water matrix with nitrate, carbonate, and chloride [24]. However, these materials were tested in idealized conditions with minimal competing ions, whereas the Fe–Mn nanocomposite in this study maintained efficient arsenic removal in real groundwater, where competing ions are naturally present.

When assessing adsorption efficiency in real groundwater, the Fe–Mn nanocomposite demonstrated superior performance to certain Fe–Mn-based adsorbents. For example, granular activated carbon Fe–Mn binary oxide (GAC-FMBO) exhibited a breakthrough volume of only 83 BV under high NOM and phosphate concentrations [16], while our nanocomposite reached 587 BV under similar complex conditions, suggesting improved selectivity and resilience. Furthermore, the HZO@D201 nanocomposite [33] reached 600 BV under groundwater-simulated matrices, which is comparable to the 587 BV obtained in our study, reinforcing the robust performance of our material.

When operated with spiked tap water containing As(III), the FMBO nanocomposite developed in this work achieved 1750 BV before breakthrough, exceeding many reported values under real-like conditions and underscoring the scalability potential of the developed adsorbent. Some materials, such as FMBO-diatomite composites, have shown extremely high BVs (e.g., 7000 BV after 15 regeneration cycles [35]) but were tested in anaerobic groundwater, where redox conditions may enhance adsorption by minimizing the competition from oxidized species. Others, like the FMBO-impregnated nylon 6 fiber (IMBNP-nylon 6), demonstrated up to 21,000 BV in RO water [27], although the absence of naturally occurring ions raises concerns about real-world applicability.

Overall, although higher breakthrough volumes have been reported in synthetic or spiked systems, our Fe–Mn nanocomposite displayed excellent performance in both real and simulated matrices, indicating its strong practical potential for sustainable arsenic removal.

#### Modeling of Arsenic Adsorption in Fixed-Bed Columns

**Figure 3:**
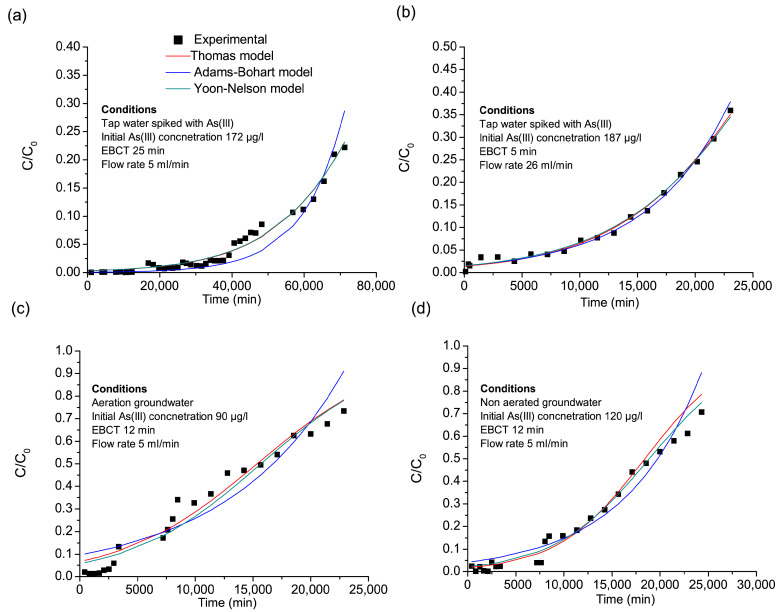
Fitting of nonlinear Thomas, Adams–Bohart, and Yoon–Nelson models to the experimental data for arsenic adsorption in the fixed-bed column studies of the Fe-Mn nanocomposite. (**a**) Column I, (**b**) Column II, (**c**) Column III, and (**d**) Column IV (raw data supplied in Table S3 of the Supplementary Information).

The data obtained from the fixed-bed column experiments were modeled using three widely applied kinetic models: Thomas, Adams–Bohart, and Yoon–Nelson (Figure 3). These models provide insights into adsorption capacity, rate constants, and breakthrough behavior under different operational conditions (Table 8).

All three models—Thomas, Adams–Bohart, and Yoon–Nelson—exhibited high R^2^ values (≥0.97 for most columns), confirming their suitability for predicting arsenic adsorption and breakthrough behavior. However, each model describes different aspects of the adsorption process, offering a complementary perspective on the adsorption dynamics. The Thomas model is commonly used to estimate the maximum adsorption capacity (q_t_) and is based on the assumption that adsorption follows Langmuir kinetics, with no axial dispersion and mass transfer limitations at the solid–liquid interface. The Adams–Bohart model is particularly useful for describing the initial phase of the breakthrough curve, assuming that adsorption depends on both the adsorbate concentration and the number of available adsorption sites. The Yoon–Nelson model simplifies the breakthrough curve analysis by estimating the time required for 50% breakthrough of the adsorbate, making it valuable for assessing the column exhaustion time [47,48,49].

Influence of Flow Rate and EBCT on Adsorption Performance: The flow rate and empty bed contact time (EBCT) are critical parameters affecting arsenic removal efficiency in fixed-bed column studies. Column I and Column II, which treated tap water spiked with As(III), operated at different flow rates and EBCTs (25 and 5 min, respectively), providing insight into the impact of contact time on adsorption efficiency. The results indicate that Column II exhibited faster adsorption kinetics than Column I, as reflected in its higher Thomas rate constant (K_Th_ = 0.000845 L/mg min) compared to Column I (K_Th_ = 0.0003765 L/mg min). This suggests that Column II facilitated a more rapid mass transfer of arsenic onto the Fe–Mn nanocomposite, likely due to the higher flow rate reducing diffusion limitations. The Adams–Bohart model supports this observation, as Column II had a higher K_AB_ (0.000747 L/mg min) than Column I (0.000489 L/mg min), indicating that Column II reached equilibrium faster due to a higher adsorption rate. The Yoon–Nelson model further reinforces this trend, with Column II exhibiting a shorter breakthrough time (τ = 27,248 min) than Column I (τ = 90,014 min), demonstrating that Column I retained arsenic for a longer duration, albeit at a slower rate. Although Column I has a lower q_t_ than Column II, it should be born in mind that it successfully treated more As-contaminated water (1750 BV) than Column II (1600 BV) (Figure 3a,b), such that the longer ECBT is a slower but more economic solution. Yunnen et al. [50] also found that the rate constant (K_Th_) increases as the flow rate rises, whereas it decreases when the initial arsenic concentration increases. However, the maximum adsorption capacity (q_t_) follows the opposite trend, increasing with higher initial arsenic concentrations but decreasing as the flow rate increases. This behavior can be attributed to the strong adsorption driving force created by the concentration gradient between the arsenic ions in solution and those on the adsorbent surface. As a result, the column’s performance improves under conditions where a higher initial arsenic concentration is present, allowing for more effective adsorption. Overall, these findings highlight the compromise between faster adsorption kinetics in Column II and the slower but prolonged adsorption process in Column I, emphasizing the need to optimize flow rates and EBCTs to balance adsorption efficiency, treated BVs, and the breakthrough time for practical applications.

Impact of Water Matrix on Adsorption Efficiency (Column III and Column IV): The complexity of real groundwater composition was evident in the adsorption behavior observed in Column III and Column IV, with untreated and aerated groundwater, respectively. The breakthrough curves indicated that arsenic removal was significantly lower in the groundwater than in the tap water, with breakthrough occurring at 365 BV in Column III and 587 BV in Column IV. The Thomas model confirmed this trend, showing lower adsorption capacities in the groundwater columns (q_t_ = 0.252 mg/g for Column III and 0.405 mg/g for Column IV), suggesting that competing ions (primarily phosphate) reduced arsenic adsorption efficiency. The higher Thomas rate constant (K_Th_) in the groundwater columns further indicated a faster initial adsorption rate, but due to rapid saturation, arsenic broke through the column earlier. Similarly, the Adams–Bohart model showed higher K_AB_ values in the groundwater columns, indicating rapid initial adsorption, but the limited availability of active adsorption sites resulted in early saturation. The Yoon–Nelson model also reflected shorter adsorption half-times (τ) in the groundwater columns, confirming that the groundwater columns had a considerably shorter operative life than the tap water columns. These findings demonstrate that groundwater chemistry plays a crucial role in determining adsorption efficiency, requiring additional considerations when applying Fe-Mn-based nanocomposites in real-world treatment systems.

Effect of Aeration on Arsenic Removal Efficiency (Column III vs. Column IV): To investigate the impact of aeration as a pre-treatment strategy, the performance of Column III (aerated groundwater) was compared to that of Column IV (untreated groundwater). The results showed that aeration did not enhance arsenic adsorption efficiency. In fact, a lower adsorption capacity (q_t_ = 0.252 mg/g) and reduced breakthrough volume (BV) were observed in Column III. This may be attributed to the removal of Fe^2+^ through aeration, which reduced the co-precipitation potential that could otherwise aid arsenic removal. While changes in arsenic speciation or competitive adsorption cannot be ruled out, it is unlikely that precipitates blocked adsorption sites on the FMBO since they were retained by the sand filter prior to column loading. However, as redox conditions and arsenic speciation were not measured at the column inlet, the exact mechanism remains uncertain. These findings highlight the need to evaluate pre-treatment steps like aeration in conjunction with specific adsorbents and water chemistry. Further testing at the pilot scale is planned to better understand these effects under representative field conditions.

### 3.4. Pilot Study

**Figure 4:**
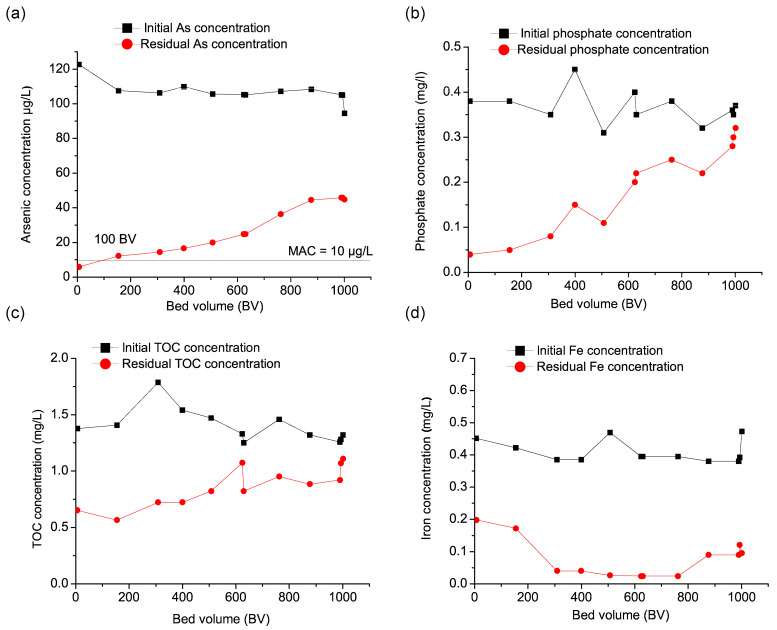
(**a**) Breakthrough curves in Pilot A of (**a**) arsenic, (**b**) phosphate, (**c**) TOC, and (**d**) iron. Initial As concentration 115 ± 6.4 µg/L, phosphate 0.36 ± 0.04 mg/L, TOC 1.4 0 ± 0.17 mg/L, Fe 0.41 ± 0.04 mg/L, flow rate 40 L/h (0.67 L/min), EBCT 5 min (raw data supplied in Table S4 of the Supplementary Information).

**Figure 5:**
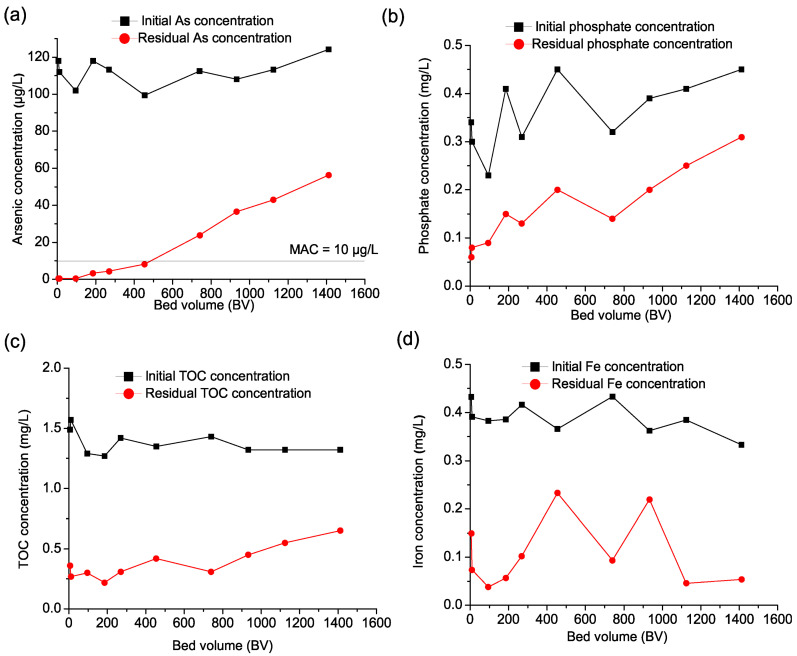
Breakthrough curves in Pilot B for (**a**) arsenic, (**b**) phosphate, (**c**) TOC, and (**d**) iron. Initial As concentration 115 ± 6.4 µg/L, phosphate 0.33 ± 0.11 mg/L, TOC 1.38 ± 0.10 mg/L, Fe 0.39 ± 0.03 mg/L, flow rate 22 L/h (0.367 L/min), EBCT 15 min (raw data supplied in Table S5 in the Supplementary Information).

After conducting batch and laboratory-scale column experiments, the performance of the Fe–Mn-based material for arsenic removal from groundwater containing arsenic (Table 1) was evaluated in a pilot study under two different operating conditions. In Pilot A, the system operated at a flow rate of 40 L/h (0.67 L/min), while in Pilot B, the flow rate was 22 L/h (0.367 L/min) (Table 5). Figure 4 and Figure 5 present the breakthrough curves obtained for these different flow rates.

The results presented in Figure 4a indicate that the Fe–Mn nanocomposite initially exhibited high efficiency (95.1%) in removing arsenic from the groundwater. As filtration progressed, efficiency declined, leading to breakthrough after 100 bed volumes (BVs) of treated water, indicating a progressive saturation of adsorption sites. Beyond this point, a more pronounced decrease was observed, with removal efficiency further reducing to 52.7% by the end of the experiment. As previously discussed in Section 3.2, this reduced operational lifespan of the Fe–Mn nanocomposite can be attributed to adsorption site saturation caused by the presence of competing anions, particularly phosphate and natural organic matter.

To better understand this effect, the concentrations of these anions were also monitored during the filtration process. Figure 4b illustrates phosphate removal, showing an initial efficiency of 89.47%, which gradually decreased to 13.5% over time. The sharp decline in phosphate removal suggests strong competition between phosphate and arsenic for adsorption sites, which likely contributed to the decreased arsenic removal efficiency. Given that phosphates are known to have a high affinity for iron-based adsorbents, their presence in groundwater significantly impacts the adsorption of arsenic, as observed in previous studies [11,12,16,17]. Additionally, Figure 4c presents the behavior of total organic carbon (TOC) removal during filtration. The Fe–Mn nanocomposite initially exhibited a TOC removal efficiency of 59.9%, which fluctuated but remained relatively high during the early stages of filtration. However, as filtration continued, TOC removal efficiency declined steadily, reaching 15.9% by the end of the experiment. The reduced removal efficiency suggests that organic matter interacts with the adsorbent, possibly blocking active sites and further decreasing its capacity for arsenic adsorption [51]. Given its elevated concentration in the investigated groundwater (Table 1), iron levels were also monitored during the pilot experiments (Figure 4d). The results indicate that iron removal began at 56.2% in the early filtration phase, with a notable improvement as filtration progressed, reaching 89.6% and peaking at 94.5%. As the experiment continued, iron removal efficiency remained stable at 93.7% for a significant portion of the operation. However, a gradual decline was observed later in the experiment. This suggests that adsorption sites on the Fe–Mn nanocomposite became saturated over time, leading to reduced removal efficiency.

The results presented in Figure 5a indicate that at a slower filtration rate, the Fe–Mn nanocomposite exhibited prolonged arsenic removal efficiency compared to experiments conducted at higher flow rates. Initially, arsenic removal was highly effective, achieving an efficiency above 99%, which remained stable for an extended duration due to the reduced filtration velocity. This slower flow rate contributed to a delayed breakthrough, occurring at approximately 475 BV, significantly later than in Pilot A, where breakthrough was observed after only 100 BV. The longer empty bed contact time (EBCT) likely enhanced arsenic adsorption by allowing better interaction between the Fe–Mn nanocomposite and the contaminant, thereby extending the operational lifespan of the adsorbent. As the adsorption sites gradually became saturated, arsenic removal efficiency began to decline, dropping to 91.8% mid-operation and further decreasing to 54.7% by the end of the experiment. This trend is consistent with findings by Chang et al. [23], who investigated arsenic removal from groundwater using diatomite-FMBO. Their study also observed a significant increase in breakthrough effluent volume with longer EBCTs, which was attributed to the limited intra-particle diffusion of the adsorbate into the adsorbent’s pores. In their experiments, breakthrough occurred at 180, 450, and 550 BV for EBCTs of 5, 10, and 20 min, respectively.

As shown in Figure 5b, phosphate removal followed a similar trend. The initial efficiency was 82.4%, which gradually decreased over time. Unlike in the previous experiment, where phosphate removal dropped sharply after 500 BV, in this case, the efficiency declined more gradually, reaching 31.1% at the later stages of operation. The extended contact time appeared to mitigate some of the negative competitive adsorption effects, allowing the Fe–Mn nanocomposite to sustain phosphate removal for a longer period. However, despite this improvement, the competition between arsenic and phosphate remained a limiting factor for arsenic removal, reducing the overall adsorption efficiency as filtration progressed. Figure 5c presents TOC removal trends under the slower filtration rate. The initial TOC removal efficiency was 82.8%, fluctuating but remaining relatively high during the early stages of filtration. As with arsenic and phosphate, TOC removal efficiency gradually decreased over time, dropping to 50.8% at the end of the experiment. The lower filtration velocity allowed for more effective organic matter adsorption, reducing the fouling effects that were more prominent in the higher flow rate experiment. Nonetheless, as filtration continued, TOC still interfered with adsorption, likely blocking the active sites and further contributing to the decrease in arsenic removal efficiency. As shown in Figure 5d, iron removal began at 65.5%, and as the experiment continued, iron removal efficiency exhibited fluctuations between 39.5% and 88.2%, ultimately reaching 83.9% by the end of the study, which suggests that adsorption sites on the Fe–Mn nanocomposite became saturated over time.

Compared to the higher filtration rate, Pilot B demonstrated enhanced arsenic removal efficiency and a delayed breakthrough point, confirming that a slower flow rate improves adsorption performance. The longer residence time facilitated better mass transfer and prolonged the Fe–Mn nanocomposite’s operational lifespan. However, despite this advantage, the presence of phosphate and TOC still contributed to performance decline, emphasizing the importance of pre-treatment strategies to minimize competitive adsorption effects.

#### 3.4.1. Modeling of Arsenic Adsorption Under Realistic Treatment Conditions

**Figure 6:**
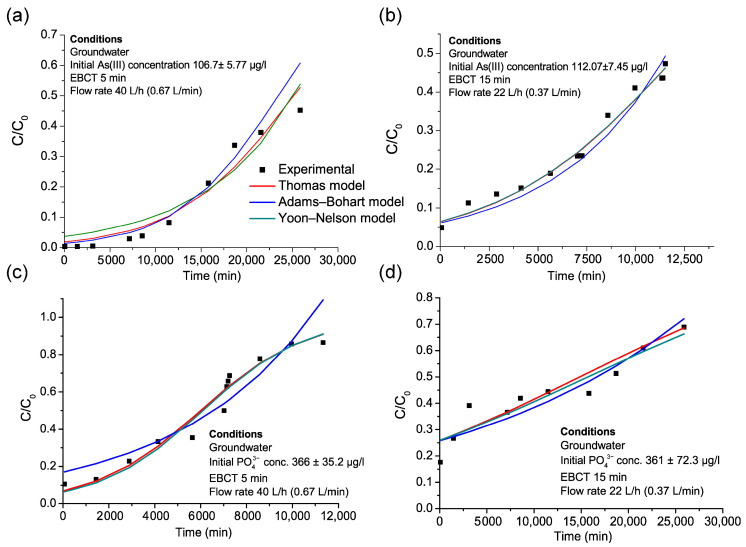
Nonlinear fitting of the Thomas, Adams–Bohart, and Yoon–Nelson models to the data from the pilot: (**a**) Pilot A As removal, (**b**) Pilot B arsenic removal, (**c**) Pilot A phosphate removal, (**d**) Pilot B phosphate removal.

To further assess the adsorption capacity of the adsorbent for arsenic removal under realistic conditions and to evaluate its performance across different operational parameters (including varying flow rates, EBCTs, and bed heights) (Table 8), data from the two pilot-scale investigations were modeled using the Thomas, Adams–Bohart, and Yoon–Nelson models (Figure 6, Table 9). Given that phosphate is recognized as the primary competitor for arsenic, its adsorption capacity on the Fe–Mn nanocomposite was also evaluated.

The Thomas model results show that the theoretical capacity (q_t_) was higher for arsenic in Pilot A (0.551 mg/g) than in Pilot B (0.417 mg/g), suggesting that higher flow rates enhance the initial adsorption capacity. However, the breakthrough time was significantly longer in Pilot B, as confirmed by the Yoon–Nelson model, which predicted breakthrough times of 23,463 min in Pilot B compared to 12,197 min in Pilot A. This was consistent with the observed breakthrough volumes of 100 vs. 450 BV (Figure 5). This confirms that a lower flow rate and longer EBCT improve the operational lifespan, delaying arsenic breakthrough and increasing adsorption stability, as was also observed in the results of the column experiments (Figure 3). The Adams–Bohart model shows that the saturation adsorption capacity (N_o_) for arsenic was 327 mg/L in Pilot A and 216 mg/L in Pilot B, indicating that arsenic uptake was more effective in the higher flow rate system. However, the rate constant (K_AB_) was higher in Pilot A (0.00171 L/mg min) than in Pilot B (0.000924 L/mg min), reinforcing that higher flow rates lead to faster adsorption kinetics but also faster saturation of the adsorbent (Table 8).

Phosphate is recognized as a strong competitor for arsenic adsorption due to its higher affinity for iron-based adsorbents. The modeling results confirm that phosphate adsorption followed a similar trend to arsenic but with a higher overall adsorption capacity. The Thomas model shows that the phosphate adsorption capacity was 0.926 mg/g in Pilot A and 0.823 mg/g in Pilot B, significantly higher than for arsenic adsorption. This confirms that phosphate strongly competes for adsorption sites, reducing arsenic removal efficiency wherever it is present [28,52,53]. The Yoon–Nelson model results indicate that phosphate adsorption lasted longer in Pilot B (7291 min) than in Pilot A (6091 min), further suggesting that a lower flow rate and higher bed volume improve phosphate retention. However, the rate constant (K_YN_) was higher in Pilot A (0.000445 min^−1^) than in Pilot B (0.000128 min^−1^), indicating that phosphate adsorption was faster under higher flow conditions but less sustainable over time. The Adams–Bohart model also supports this trend, showing that the adsorption capacity coefficient (N_o_) for phosphate was much higher than that for arsenic (792 mg/L in Pilot A and 761 mg/L in Pilot B). However, the rate constant (K_AB_) for phosphate was significantly lower than that for arsenic, confirming slower initial adsorption kinetics despite the higher overall capacity. The competition between phosphate and arsenic is further evident from the greater phosphate adsorption capacities in both pilots. In Pilot B, where phosphate adsorption lasted longer, arsenic removal efficiency was lower, suggesting that phosphate outcompetes arsenic at a lower flow rate and higher bed height (Table 8). These findings suggest that optimizing operational parameters—such as flow rate, EBCT, and, potentially, a pre-treatment for phosphate removal—can significantly enhance arsenic removal efficiency in real-world applications and prolong the lifespan of the adsorbent.

#### 3.4.2. Arsenic Speciation and Removal Mechanism

To better understand the arsenic removal mechanism of the Fe–Mn nanocomposite under realistic treatment conditions, arsenic speciation was performed on selected samples prior to breakthrough during the pilot-scale testing. As shown in Figure S5, As(III) was the dominant species in the raw groundwater, accounting for more than 90% of total arsenic. Following treatment with the FMBO nanocomposite, a partial conversion of As(III) to As(V) was observed. This suggests that the material possesses inherent oxidative capacity, likely due to the presence of Mn(IV) species embedded within the nanocomposite matrix. Importantly, despite only partial oxidation, the FMBO nanocomposite demonstrated high removal efficiency for As(III), highlighting its strong affinity for this more mobile and toxic species.

These findings support a dual mechanism of arsenic removal: (i) oxidation of As(III) to As(V), followed by adsorption of As(V) onto Fe-based sites, and (ii) direct adsorption of As(III) onto the active surface sites of the nanocomposite. The combined oxidative and adsorptive properties of the FMBO material enable efficient removal of both As species, even under real-world conditions. This behavior has been consistently reported in the literature for Fe–Mn-based adsorbents [11,13,14,21,22,23,24,25,26] and reinforces the potential of this material for practical arsenic remediation applications.

### 3.5. Comparison Between Batch Experiments and Pilot-Scale Studies

To evaluate whether a correlation exists between the batch adsorption experiments, fixed-bed column studies, and pilot-scale investigations, adsorption capacities were compared across the different scales while focusing only on real groundwater conditions (Table 10). The batch adsorption experiments, modeled using the Langmuir isotherm, predicted a maximum adsorption capacity (q_max_) of 4.71 mg/g for arsenic-contaminated groundwater. In contrast, adsorption capacities obtained from the Thomas model in the fixed-bed column studies were 0.252 mg/g in Column III (aerated groundwater) and 0.405 mg/g in Column IV (non-aerated groundwater). In the pilot-scale study, the adsorption capacities ranged from 0.551 mg/g in Pilot A to 0.417 mg/g in Pilot B.

The modeled adsorption capacity in the batch experiments was significantly higher than that in the column and pilot studies, which was expected since batch systems provide sufficient equilibrium time and significantly higher theoretical maximum loads, allowing the adsorbent to reach its maximum adsorption potential. In contrast, flow-through systems operate under dynamic conditions, where adsorption efficiency is influenced by flow rate, EBCT, and competitive adsorption effects. Among the fixed-bed column experiments and pilot-scale adsorption studies, Column III, Column IV, and Pilot B share similar EBCT values (~15 min), which contributes to their comparable adsorption performance. Notably, the adsorption capacity in Pilot B (0.243 mg/g) closely aligns with that in Column IV (0.238 mg/g), indicating a strong correlation between fixed-bed column experiments and pilot-scale adsorption studies. Additionally, the breakthrough point for Pilot B (475 BV) is similar to that of Column IV (587 BV), further supporting this relationship.

The impact of flow rates, EBCTs, and bed depths is evident when comparing adsorption behavior across different setups. Pilot A, which had a shorter EBCT (~5 min), exhibited lower arsenic adsorption and an earlier breakthrough (100 BV), confirming that a shorter contact time reduces adsorption efficiency. Meanwhile, the longer EBCT in Pilot B (15 min) resulted in extended adsorption performance, similar to what was observed in Columns III and IV. Additionally, the greater bed height in the pilot-scale study than in the column experiments likely contributed to prolonged breakthrough times.

Overall, these findings highlight the importance of continuous-flow experiments in assessing the performance of new adsorbents. The investigations using real groundwater samples confirmed that the batch experiments tend to overestimate adsorption capacity, whereas fixed-bed column studies provide more reliable predictions for pilot-scale adsorption performance. The strong agreement between Column IV and Pilot B suggests that well-designed fixed-bed column studies can effectively simulate significantly larger real-world pilot-scale conditions, although it should be noted that even the column experiments in this work are about an order of magnitude larger than many reported in the literature. The results from the experiments using raw groundwater emphasize the need to optimize operational parameters and implement pre-treatment strategies for competing anions to enhance arsenic removal efficiency in large-scale applications, as well as the benefits of optimizing adsorbents for more selective arsenic removal, especially over phosphate [54]. Selective adsorption can be enhanced by exploiting the physicochemical differences between arsenic and phosphate, particularly in complexation behavior, Lewis acid–base interactions, charge density, and molecular size or geometry. For example, transition metal–chitosan complexes, such as Cu(II)- and Fe(III)-chitosan, have shown promise in preferentially adsorbing arsenate over phosphate by creating binding sites that capitalize on these differences in physicochemical properties [55,56,57]. Beyond adsorbent modification, several pre-treatment strategies can be employed to mitigate phosphate interference, including selective phosphate removal via ion exchange resins, chemical precipitation, and membrane filtration techniques that discriminate based on charge or molecular size. While these strategies were not explored in the present study, future research will focus on engineering FMBO nanocomposites with enhanced selectivity and/or integrating them with phosphate-targeting pre-treatment steps to improve arsenic removal performance under realistic water matrix conditions.

### 3.6. Regeneration and Reusability of the FMBO Nanocomposite

**Figure 7:**
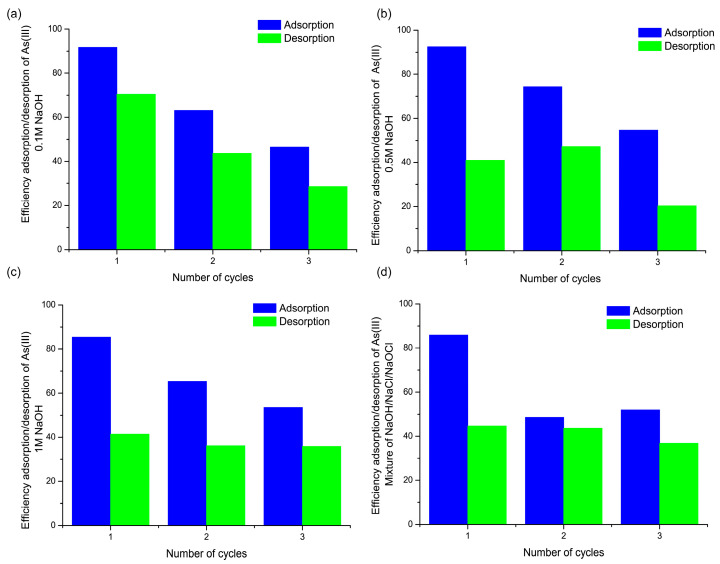
Regeneration and reusability of the FMBO nanocomposite over three consecutive adsorption–desorption cycles using different regenerants: (**a**) 0.1 M NaOH, (**b**) 0.5 M NaOH, (**c**) 1 M NaOH, and (**d**) a mixed solution of NaOH–NaCl–NaOCl. Adsorption conditions: initial As(III) concentration = 0.2 mg/L; FMBO dose = 0.5 g; pH = 7.0 ± 0.2; contact time = 2 h per cycle.

The reusability of the FMBO nanocomposite was assessed over three successive adsorption–desorption cycles using different regenerants, with the results presented in Figure 7. Among the tested eluents, 0.1 M NaOH (Figure 7a) demonstrated the most favorable balance between adsorption and desorption efficiencies across cycles. Initial adsorption efficiency was above 90%, with desorption around 65%, and both declined gradually over the cycles yet retained more than 50% efficiency by the third cycle.

When higher NaOH concentrations were applied (0.5 M and 1 M, Figure 7b,c), no significant improvement was observed. Instead, the adsorption efficiency dropped faster with repeated cycles, suggesting that stronger alkaline conditions may compromise the stability of the adsorbent or cause the irreversible binding of arsenic species.

Interestingly, the regenerant mixture containing NaOH, NaCl, and NaOCl (Figure 7d) showed the highest initial desorption efficiency (~85%), but the adsorption capacity dropped significantly in the second and third cycles, indicating potential oxidative degradation or the structural alteration of the active sites.

These findings suggest that mild alkaline conditions (0.1 M NaOH) offer the best compromise between efficient regeneration and the preservation of adsorption capacity. While further optimization and testing under continuous-flow conditions are warranted, these preliminary results confirm the potential of the FMBO nanocomposite for multiple reuses in water treatment applications. Moreover, to ensure the overall environmental safety and sustainability of the process, it is essential to consider appropriate end-of-life strategies for the exhausted material [58,59].

Although regeneration can extend the service life of the adsorbent, complete saturation is ultimately inevitable. To ensure environmental safety and sustainability, it is essential to implement appropriate end-of-life (EoL) strategies for the exhausted FMBO nanocomposite [58]. In addition to conventional landfill disposal—provided that the leaching of iron and manganese is strictly controlled—more sustainable approaches should be explored [59]. Solidification/stabilization (S/S) with cement or quicklime can effectively immobilize contaminants and allow the safe incorporation of spent adsorbents into construction materials. Reuse in the production of porous insulation or sound-absorbing materials via freeze-casting techniques has also shown promise [58]. Moreover, resource recovery processes such as acid leaching and selective precipitation could enable the extraction and reuse of iron and manganese in various industrial sectors, including metallurgy, pigment manufacturing, and battery production. Thermal treatments like pyrolysis or calcination may transform spent FMBO into catalytically active materials for advanced oxidation processes.

Future work should therefore adopt a life cycle perspective that integrates these EoL strategies into the overall assessment of FMBO-based technologies. Such a systems-level approach evaluating not only adsorption efficiency and regeneration potential but also disposal, recovery, and repurposing routes is essential to ensure both environmental sustainability and economic feasibility in real-world applications.

### 3.7. Cost Analysis and Practical Feasibility

To assess the economic viability of the FMBO nanocomposite, a preliminary cost analysis was performed by estimating the material cost required to treat 1 m^3^ of arsenic-contaminated groundwater (C_0_ ≈ 100 µg/L). Based on the pilot-scale results, the required mass of the FMBO nanocomposite ranged from 0.182 kg (Pilot A) to 0.240 kg (Pilot B) per cubic meter of water treated. Assuming a conservative production cost of 12 EUR/kg, the total adsorbent cost per m^3^ of treated water was estimated to range between EUR 2.18 and EUR 2.88. This is significantly lower than the cost of widely used commercial iron-based adsorbents such as Bayoxide E33, which has a reported market price of 35 EUR/kg and a treatment cost of ~10.85 EUR/m^3^ under comparable conditions [60]. These findings emphasize the scalability and market potential of the FMBO nanocomposite as a sustainable and economically viable solution for arsenic removal from drinking water.

## 4. Conclusions

This study presents a comprehensive evaluation of an Fe–Mn-based nanocomposite (FMBO) for arsenic removal across batch, column, and pilot-scale systems using both synthetic and real groundwater matrices. The nanocomposite demonstrated promising performance, with batch adsorption tests indicating high removal efficiencies for both As(III) and As(V), reaching up to 6.25 mg/g. However, fixed-bed column and pilot-scale experiments revealed more realistic adsorption capacities (0.405–0.551 mg/g), emphasizing the importance of dynamic testing for scaling applications. The similarity in breakthrough behavior between Column IV and Pilot B highlighted that well-designed column experiments can reliably predict performance under field conditions.

A key limitation identified was the strong competitive interference from phosphate, which significantly reduced arsenic uptake. While this study focused on evaluating FMBO performance under realistic water compositions, future research should investigate strategies to mitigate such interference, including adsorbent surface modification for improved selectivity or the integration of a phosphate-removal pre-treatment step.

Preliminary regeneration studies further demonstrated the potential for FMBO reuse, with 0.1 M NaOH identified as the most effective regenerant, maintaining over 50% removal efficiency after three cycles. Although more extensive continuous regeneration tests are needed, these findings suggest promising operational stability.

In addition to regeneration performance, attention must be given to the safe and sustainable end-of-life management of the exhausted adsorbent. While not the primary focus of this study, preliminary investigations have considered strategies such as solidification/stabilization and reuse in construction materials to prevent secondary contamination from leached metals. Future work will further explore these approaches, including environmental risk assessment and life cycle analysis, to ensure responsible disposal and to support circular economy principles.

Overall, the results underscore the importance of bridging laboratory-scale findings with real-world testing and provide a strong foundation for the continued development and optimization of FMBO-based technologies for sustainable arsenic removal in drinking water treatment systems.

## Figures and Tables

**Table 1:** Adsorption performance of Fe–Mn-based adsorbents in a batch system.

Fe–Mn-Based Adsorbents	Initial As Concentration (mg/L)	Water Matrix	pH	Adsorption Capacity in Batch System (mg/g)		References
				As(III)	As(V)	
Fe–Mn binary oxide	1–200	Synthetic	5	69.8	133	[21]
Magnetite coated with FMBO	0.2–50	Synthetic	7.0	55.9	54.1	[22]
Diatomite coated with Fe–Mn binary oxide	0.05–20	Synthetic	7.0	1.68	-	[23]
Macroporous anion exchanger-supported Fe–Mn binary oxide	1–50	Synthetic water	7.0	44.9	13.17	[24]
Starch-FMBO	0–300	Synthetic		161	-	[25]
Gelatin-FMBO	0–300			141	-	[25]
CMC-FMBO	0–300			104	-	[25]
Nanoscale Fe–Mn binary oxides loaded on zeolite (NIMZ)	2–100	Synthetic	7.0	47	49	[26]
Iron–manganese binary oxide nanoparticles on nylon 6 fiber	1–100	Synthetic	7.0	134	-	[27]
Biochar coated with FMBO	0.01–10	Synthetic	7.0	14.4	12.2	[9]
GAC-FeMn	0.1–1	Synthetic	7.0	2.30	2.87	[16]
Chitosan-FMBO	0–24	Synthetic	7.0	54.2	-	[28]
Chitosan-FeMn	0.1–1	Synthetic	7.0	3.91	3.89	[29]
PET-FMBO	0.1–10	Synthetic	7.0	8.74	13.3	[13]
PE-FMBO	0.1–10	Synthetic	7.0	5.29	5.37	[13]
Graphene oxide chitosan-coated FMBO (Fe/Mn GOCS)	5–300	Synthetic	7.62	109	-	[11]
Fe/Mn-C-layered double-hydroxide composite	5–100	Synthetic	-	41.9	33.6	[30]
FMBO nanocomposite	0.1–10	Synthetic	7.0	6.2	-	This study

**Table 2:** Characteristics of the investigated water matrices.

Parameter	Water		
	Groundwater	Aerated Groundwater	Spiked Tap Water
pH	7.73 ± 0.05	7.81 ± 0.05	7.51 ± 0.04
Conductivity (µS/cm)	514 ± 43	466 ± 12	490 ± 22
Turbidity (NTU)	1.55 ± 0.29	0.70 ± 0.05	0.31 ± 0.07
TOC (mg/L)	1.38 ± 0.10	1.53 ± 0.05	1.460 ± 0.05
Arsenic (μg/L)	115 ± 6.4	90.0 ± 18.7	172 ± 20
Iron (μg/L)	395 ± 28.1	5.36 ± 3.55	26.04 ± 2.20
Manganese (μg/L)	54 ± 4.67	6.35 ± 8.52	3.22 ± 4.10
Ammonium (mg N/L)	0.44 ± 0.09	0.228 ± 0.12	0.454 ± 0.01
Nitrate (mg N/L)	1.57 ± 0.14	0.67 ± 0.11	0.05 ± 0.01
Orthophosphate (mg PO_4_/L)	0.323 ± 0.11	0.075 ± 0.02	0.024 ± 0.01
Chloride (mg Cl/L)	3.77 ± 3.19	0.673 ± 0.19	27.25 ± 0.01

**Table 3:** Isotherm models used for fitting the adsorption data obtained for arsenic adsorption in batch systems.

Model	Equation	Parameters
Freundlich	$q_{e}=K_{F}C_{e}^{n_{F}}$	qₑ—Adsorbed amount at equilibrium (mg/g), Cₑ—Equilibrium concentration (mg/L), K_F_—Freundlich adsorption constant [(mg/g)(L/mg)^(1/n)], n_F_—Freundlich exponent
Langmuir	$q_{e}=\frac{q_{max}K_{L}C_{e}}{1+K_{L}C_{e}}$	q_e_—Adsorbed amount at equilibrium (mg/g), Cₑ—Equilibrium concentration (mg/L), q_max_—Maximum adsorption capacity (mg/g), K_L_—Langmuir constant (L/mg)

**Table 4:** Column adsorption models used for fitting adsorption data from column and pilot studies.

Models	Equation	Parameters
Thomas	$\frac{c_{t}}{c_{0}}=\frac{1}{1+exp\left(k_{Th}q_{0}\frac{m}{Q}-c_{0}t\right)}$	*c*_t_: Concentration at time (mg/L), c_0_: Initial concentration (mg/L), *k*_Th_: Thomas rate constant (L/min·mg), *q*_0_: Maximum adsorption capacity (mg/g), *m*: Mass of adsorbent (g), *Q*: Flow rate (L/min), *t*: Time (min)
Adams–Bohart	$\frac{c_{t}}{c_{0}}=\exp\left(k_{AB}c_{0}t-k_{AB}N_{0}\frac{L}{u}\right)$	*c_t_*: Concentration at time (mg/L), *c*_0_: Initial concentration (mg/L), *k_AB_*: Adams–Bohart rate constant (L/mg·min), *N*_0_: Saturated adsorption capacity (mg/L), *L*: Packed bed length (cm), *t*: Time (min), *u*: Linear velocity (cm/min)
Yoon–Nelson	$\frac{c_{t}}{c_{0}}=\frac{1}{1+\exp\;\left(k_{YN}\left(\tau -t\right)\right)}$	*c_t_*: Concentration at time (mg/L), *c*_0_: Initial concentration (mg/L), *k_YN_*: Yoon–Nelson rate constant (L/min), *τ*: Time required for 50% breakthrough (min), *t*: Time (min)

**Table 5:** Operational conditions at the pilot filtration unit with an Fe-Mn nanocomposite adsorbent.

	Filter Media Volume (L)	Mass of Media (kg)	Bed Depth (m)	Filtration Rate (m/h)	EBCT (min)	Flow Rate Q (L/h)
Pilot A	3.5	1.6	0.11	1.25	5.12	40
Pilot B	5.5	2.5	0.17	0.62	16.5	22

**Table 6:** Parameters of Freundlich and Langmuir isotherm models for arsenic adsorption on an FMBO nanocomposite.

	Freundlich Model			Langmuir Model		
Matrix Type	n_F_	K_F_ (mg/g)/(mg/L)^n^	R^2^	q_max_ (mg/g)	K_L_ (L/mg)	R^2^
As(III) spiked tap water	0.640	2.70	0.9459	6.25	0.992	0.9577
As(III) spiked groundwater	0.420	1.79	0.9349	4.63	0.343	0.9452

**Table 7:** Adsorption performance of Fe–Mn-based and similar adsorbents in continuous systems.

Fe–Mn-Based Adsorbent	Length (cm)	Diameter (cm)	Mass of Adsorbent (g)	Bed Depth (cm)	Flow Rate (mL/min)	EBCT (min)	Water Matrix	BV Before MAC Breakthrough	Ref.
Graphene oxide chitosan-coated FMBO (Fe/Mn GOCS)	3	1.6	7.67	-	1.5	-	Synthetic water matrix 10 or 50 mg/L As(III) pH 7	40 and 3	[11]
Macroporous anion exchanger-supported Fe–Mn binary oxide	13.0	1.2	-	-	-	3	Simulated water As(III) 100 µg/L; Nitrate 150 mg/L; Carbonate 200 mg/L; Chloride 300 mg/L; Sulfate 300 mg/L; pH 8.10	2300	[24]
Chitosan coated with Fe–Mn binary	32	1.9	30	25	-	10	Simulated groundwater As(III)/As(V) 233 μg/L; Nitrate 5 mg/L, Carbonate: 159 mg/L; Silicate 12 mg/L; Phosphate 0.13 mg/L; pH 7.3	500 and 3200 for As(V) and As(III)	[29]
GAC-FMBO	80	1.7	-	30	6	12	Groundwater As(III): 120 µg/L; Conduct. 678 mS/cm; DOC 2.00 mg/L; Alkalinity 7.68 mmol/L; Chloride 19.7 mg/L; Carbonate 118 mg/L; Sulfate 15.7 mg SO_4_/L; Phosphate 1.33 mg/L; Fe 35.4 µg/L Mn 21.5 µg/L; pH 8.22	83	[16]
HZO@D201 nanocomposite	13	1.2	5 mL	-	-	3	Simulated groundwater As(III) 0.1 mg/L; Magnesium 5 mg/L; Sulfate 50 mg/L; Calcium 15 mg/L; Silicate 5 mg/L; Chloride 40 mg/L; Nitrate 8 mg/L; Carbonate 150 mg/L; pH: 8.2	600	[33]
FMBO-diatomite	40	3	-	-	34	5	Spiked DI water with As(III)	4500	[34]
FMBO-diatomite	40	3	58	24	17	10	Anaerobic groundwater; As_tot_ 0.0477 mg/L; As(III) 0.03 mg/L; Turbidity 0.7 NTU; Conductivity 530 mS/cm; TOC 2.84 mg/L; 305 mg/L; Magnesium 16 mg/L; Calcium 28 mg/L; Chloride 40 mg/L; Nitrate 9.8 mg/L; Phosphor 1.21 mg/L; Mn 0.15.1 mg/L; Fe 0.257 mg/L; Nitrogen 66 mg/L; pH: 7.4	7000 BV after 15 regenerations	[35]
FMBO-impregnated nylon 6 fiber (IMBNP-nylon 6)	4	1	-	-	-	0.65	Spiked RO water with As(III) 0.1 and 0.038 mg/L; Magnesium 5 mg/L; Sulfate 50 mg/L; Calcium 15 mg/L; Silicate 5 mg/ L; Chloride 40 mg/L; Nitrate 8 mg/L; Carbonate 150 mg/L; pH: 8.2	5200 and 21,000	[27]
FMBO nanocomposite	30	2	28	20	5.2	12	Raw groundwater As_tot_ 0.115 mg/L; Orthophosphate 0.323 mg/L; Chloride 3.77 mg/L; Nitrate 1.57 mg/L; Mn 0.054 mg/L; Fe 0.395 mg/L pH: 7.73	587	This work
	60	2	60	42	5.2	25	Spiked tap water with As(III) As_tot_ 0.172 mg/L; Orthophosphate 0.024 mg/L; Chloride 27.25 mg/L; Nitrate 0.05 mg/L; Mn 0.003 mg/L; Fe 0.026 mg/L pH: 7.51	1750	

**Table 8:** Parameters of the Thomas, Adams–Bohart, and Yoon–Nelson models obtained from the modeling experimental data of As(III) adsorption on the Fe-Mn nanocomposite in fixed-bed column studies.

		Thomas Constant			Adams–Bohart			Yoon–Nelson		
	q_exp_ (mg/g)	q_t_ (mg/g)	K_Th_ (L/mg min)	R^2^	No (mg/L)	K_AB_ (L/mg min)	R^2^	k_YN_ (/min)	τ (min)	R^2^
Column I	1.02	1.34	0.0003765	0.9744	588	0.000489	0.9124	0.0000641	90,014	0.9915
Column II	1.42	1.71	0.000845	0.9920	863	0.000747	0.9909	0.000153	27,248	0.9915
Column III	0.238	0.252	0.00191	0.9824	122	0.00109	0.8754	0.000177	15,690	0.9497
Column IV	0.343	0.405	0.00183	0.9760	174	0.00104	0.9384	0.000202	18,871	0.9733

**Table 9:** Parameters of the Thomas, Adams–Bohart, and Yoon–Nelson models obtained from the modeling experimental data of arsenic and phosphate adsorption in the Fe–Mn nanocomposite pilot studies.

			Thomas Constant			Adams–Bohart			Yoon–Nelson		
		q_exp_ (mg/g)	q_t_ (mg/g)	K_Tb_ (L/mg min)	R^2^	N_o_ (mg/L)	K_AB_ (L/mg min)	R^2^	K_YN_ (min^−1^)	τ (min)	R^2^
Pilot A	Arsenic	0.337	0.551	0.00206	0.9497	327	0.00171	0.9589	0.000218	12,197	0.9802
	Phosphate	0.811	0.926	0.00118	0.9726	792	0.000448	0.8598	0.000445	6091	0.9635
Pilot B	Arsenic	0.243	0.417	0.00139	0.9485	216	0.000924	0.9502	0.000181	23,463	0.9234
	Phosphate	0.731	0.789	0.000196	0.9864	770	0.000111	0.8818	0.0000667	15,719	0.9881

**Table 10:** Comparison of the adsorption capacity of the Fe-Mn nanocomposite obtained at different scales, from batch to pilot.

Scale	Mass Adsorbent	q_exp_ (mg/g)	q (Langmuir or Thomas Model) (mg/g)	Breakthrough Point
Batch experiments	20 mg		4.71	
Column III	28 g	0.238	0.252	587
Column IV	28 g	0.343	0.405	365
Pilot A	1.6 kg	0.337	0.551	100
Pilot B	2.5 kg	0.243	0.417	475

## Data Availability

The original contributions presented in this study are included in the article/Supplementary Material. Further inquiries can be directed to the corresponding author(s).

## Supplementary Materials

The following supporting information can be downloaded at: https://www.mdpi.com/article/10.3390/nano15141104/s1: Figure S1: Correlation between q_max_ values reported in the literature and the maximum initial concentrations of the experimental isotherms. Figure S2: SEM/EDS analysis of the FMBO nanocomposite. Figure S3: XRD pattern of the FMBO nanocomposite. Figure S4: FTIR spectra of the FMBO nanocomposite. Figure S5: Distribution of inorganic arsenic species, As(III) and As(V), in groundwater and water after adsorption on the FMBO nanocomposite. Table S1: Operational conditions in lab-scale column experiments. Table S2: Residual concentrations of Fe and Mn in water after treatment with the Fe-Mn nanocomposite. Table S3: Raw data from Figure 3, columns I, II, III, and IV. Note that the initial concentrations of arsenic (C0) were subject to slight natural variation during the experiments. Table S4: Initial and residual concentrations of phosphates, total organic carbon, arsenic, and iron in Pilot A. Table S5: Initial and residual concentrations of phosphates, total organic carbon, arsenic, and iron in Pilot B.
