# Supplementary material for: From Batch to Pilot: Scaling Up Arsenic Removal with an Fe-Mn-Based Nanocomposite
Source: Nanomaterials (Basel). 2025 Jul 16;15(14):1104. doi: 10.3390/nano15141104 (PMC12300050; doi:10.3390/nano15141104)
Supplement: Supplementary file 1 [file nanomaterials-15-01104-s001.zip › nanomaterials-3736973-supplementary.pdf]

nanomaterials

MDPI

Supplementary material

From Batch to Pilot: Scaling Up Arsenic Removal with an Fe-Mn-Based Nanocomposite

From Batch to Pilot: Scaling Up Arsenic Removal with an Fe-Mn-Based Nanocomposite

Jasmina Nikić, Jovana Jokić Govedarica, Malcolm Watson *, Đorđe Pejin, Aleksandra Tubić and Jasmina Agbaba

Department of Chemistry, Biochemistry and Environmental Protection, Faculty of Sciences, University of Novi Sad, Trg Dositeja Obradovića 3, 21000 Novi Sad, Serbia; jasmina.nikic@dh.uns.ac.rs (J.N.); jovanaj@dh.uns.ac.rs (J.J.G.); djordje.pejin@dh.uns.ac.rs (Đ.P.); aleksandra.tubic@dh.uns.ac.rs (A.T.); jasmina.agbaba@dh.uns.ac.rs (J.A.)

* Correspondence: malcolm.watson@dh.uns.ac.rs

Correlation between q_{max} and initial As concentration

Figure S1 demonstrates the strong correlation between qmax values reported in the literature (taken from the papers in Table 1 of the manuscript) and the maximum As(III) concentrations investigated in the isotherm experiments of those papers. Regardless of the material and water matrix, a strong positive correlation (Adj R^2 of 0.72299) is clear. This is not unexpected but does illustrate that directly comparing q_{max} values reported in the literature, without considering the experimental conditions, does not necessarily reveal which adsorbent is the best solution for As removal from any given groundwater.

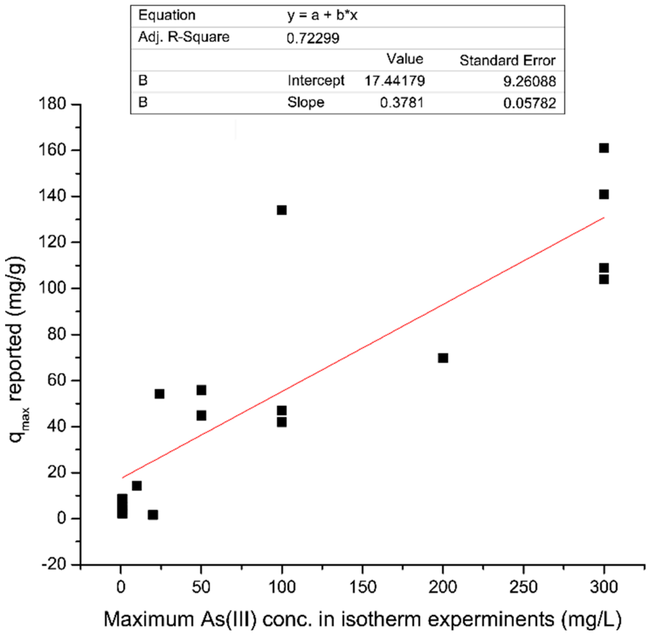

Figure S1. Correlation between q_{max} values reported in the literature and the maximum initial concentrations of the experimental isotherms.

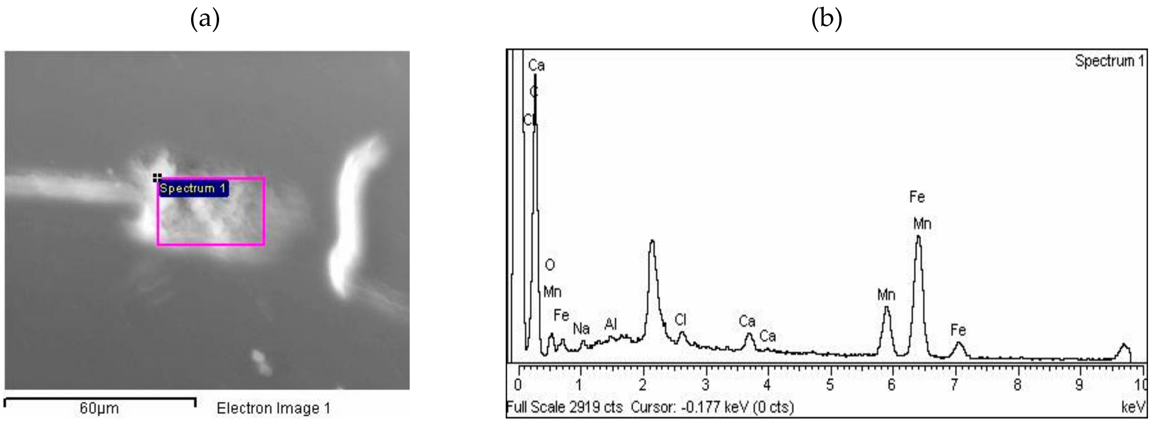

Figure S2. (a) SEM and (b) EDS analysis of the FMBO nanocomposite.

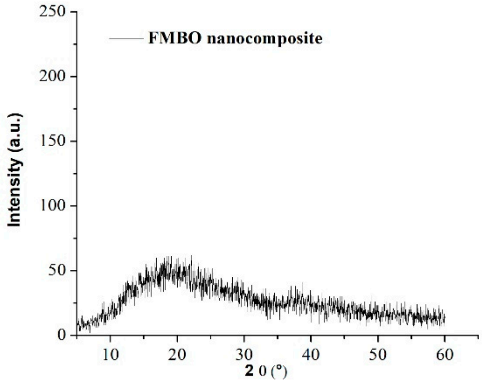

Figure S3. XRD pattern of the FMBO nanocomposite.

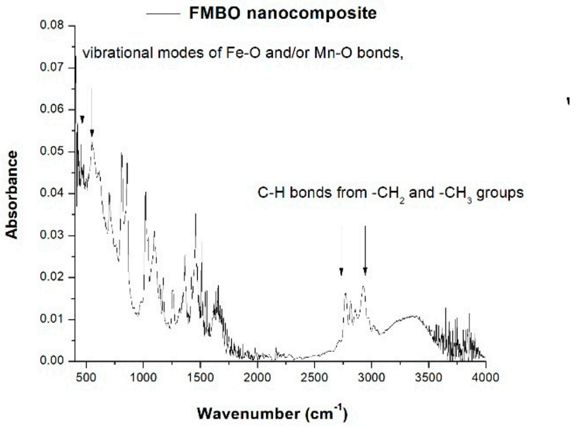

Figure S4. FTIR spectra of the FMBO nanocomposite.

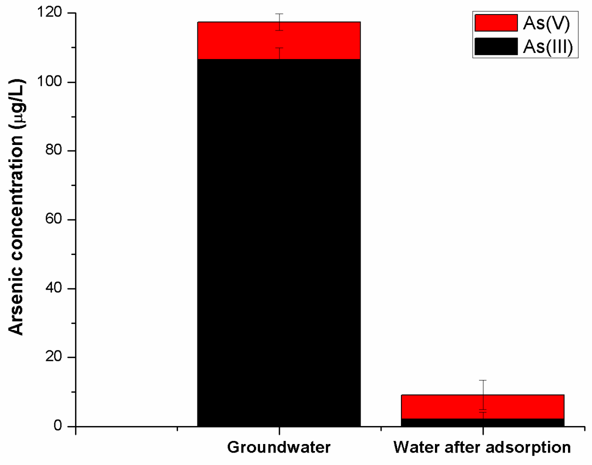

Figure S5. Distribution of inorganic arsenic species, As(III) and As(V), in groundwater and water after adsorption on the FMBO nanocomposite.

Table S1. Operational conditions in lab-scale column experiments

	Water treated	Filter media volume (ml)	Mass of media (g)	Bed depth (cm)	Filtration rate (m/h)	Flow rate (ml/min)	EBCT (min)
**Column I**	Tap water spiked with As(III)	131	60	41.7	1	5.2	25
**Column II**	Tap water spiked with As(III)	131	60	41.7	5	26.2	5
**Column III**	Aerated groundwater	62.8	28	20	1	5.2	12
**Column IV**	Groundwater	62.8	28	20	1	5.2	12

Table S2. Residual concentrations of Fe and Mn in water after treatment with the Fe-Mn nanocomposite.

**Column I**			**Column II**			**Column III**			**Column IV**		
**BV**	**Fe (μg/l)**	**Mn (μg/l)**	**BV**	**Fe (μg/l)**	**Mn (μg/l)**	**BV**	**Fe (μg/l)**	**Mn (μg/l)**	**BV**	**Fe (μg/l)**	**Mn (μg/l)**
38	39.88	43.00	23	4.42	0.28	24	3.66	1.08	24	2.6	0.4

Column I			Column II			Column III			Column IV		
**BV**	**Fe (μg/l)**	**Mn (μg/l)**	**BV**	**Fe (μg/l)**	**Mn (μg/l)**	**BV**	**Fe (μg/l)**	**Mn (μg/l)**	**BV**	**Fe (μg/l)**	**Mn (μg/l)**
154	10.39	23.00	69	5.57	0.33	48	1.19	0.33	56	1.0	0.1
200	21.60	23.42	138	4.80	0.73	111	1.62	0.24	127	0.8	0.2
250	0.98	42.12	369	4.26	0.30	159	0.93	0.36	191	0.8	0.2
300	14.36	21.00	619	4.13	0.42	239	1.04	0.26	255	0.6	0.4
354	48.95	23.00	888	4.34	0.35	239	2.87	0.52	255	3.3	1.6
415	7.10	14.00	1058	14.69	2.25	318	2.72	0.54	318	3.2	0.6
473	30.49	24.59	1231	2.27	4.11	398	2.59	0.19	446	2.4	0.5
646	5.72	0.57	1400	2.12	1.75	494	2.97	0.38	549	2.6	1.5
700	2.23	23.00	1554	2.28	4.30	605	12.16	1.52	549	3.2	3.0
762	2.10	32.00	1572	6.13	0.34	605	3.45	1.84	637	8.2	4.0
819	1.91	0.26	1818	22.09	2.95	717	4.21	1.73	812	9.2	30.0
862	1.72	25.00	2087	19.73	4.36	828	10.07	1.00	812	44.2	4.1
908	1.80	67.00	2241	1.76	0.35	876	3.43	2.51	876	34.4	3.3
962	1.65	12.00	2425	1.37	0.70	955	3.45	3.45	955	2.5	3.3
1015	1.55	13.00	2641	1.08	1.07	1019	3.85	2.13	1290	3.4	44.1
1054	1.39	24.00	2848	1.13	0.74	1083	3.39	2.84	1369	9.7	62.0
1100	22.83	52.97	2872	1.13	0.74	1162	3.70	2.14	1529	6.4	2.3
1162	42.71	31.81	3080	10.71	1.72	1242	4.75	1.19	1608	8.5	1.7
1215	11.79	34.55	3288	4.59	1.66	1322	5.37	3.28	1688	0.7	0.8
1262	13.54	43.20	3495	12.41	1.40	1529	6.44	2.30	1791	1.9	1.8
1308	16.77	27.87	23	4.42	0.28	1608	14.7	5.69	1871	1.0	1.9
1354	23.12	22.31	69	5.57	0.33				1951	4.0	1.0
1400	8.92	1.71	138	4.80	0.73						
1446	7.82	1.87	369	4.26	0.30						
1508	8.57	3.93	619	4.13	0.42						
1562	6.64	3.30	888	4.34	0.35						
1623	6.04	3.86	1058	14.69	2.25						
1681	5.35	3.54	1231	2.27	4.11						
1738	4.71	2.72	1400	2.12	1.75						
1792	4.83	2.28	1554	2.28	4.30						
1854	4.50	2.17	1572	6.13	0.34						
2186	32.15	35.38	1818	22.09	2.95						
2297	13.77	20.33	2087	19.73	4.36						
2408	17.25	17.25	2241	1.76	0.35						
2518	22.08	2.17	2425	1.37	0.70						
38	39.88	43.00	2641	1.08	1.07						
154	10.39	23.00	2848	1.13	0.74						
200	21.60	23.42	2872	1.13	0.74						
250	0.98	42.12									

	**Column I**			**Column II**			**Column III**			**Column IV**	
**BV**	**Fe (µg/l)**	**Mn (µg/l)**	**BV**	**Fe (µg/l)**	**Mn (µg/l)**	**BV**	**Fe (µg/l)**	**Mn (µg/l)**	**BV**	**Fe (µg/l)**	**Mn (µg/l)**
300	14.36	21.00									
354	48.95	23.00									
415	7.10	14.00									
473	30.49	24.59									
646	5.72	0.57									
700	2.23	23.00									
762	2.10	32.00									

Table S3: Raw data from Figure 3, columns I, II, III, and IV. Note that the initial concentrations of arsenic (C_0) were subject to slight natural variation during the experiments

	Column I			Column II	
BV	As (µg/l)	C_0	BV	As (µg/l)	C_0
0		151	0		209
38	0.11	151	23	0.50	209
154	0.14	151	69	3.97	209
200	0.14	151	138	3.20	209
250	0.17	151	369	6.95	209
300	0.18	151	619	7.18	209
354	0.25	151	888	7.24	209
415	0.10	151	1058	7.24	209
473	0.13	186	1231	5.25	209
646	2.88	186	1400	8.63	209
700	2.40	186	1554	8.50	209
762	1.45	186	1572	17.26	209
819	1.17	186	1818	12.20	259
862	1.43	186	2087	13.17	259
908	1.41	186	2241	15.02	170
962	1.54	186	2425	21.02	170
1015	3.13	186	2641	23.31	170
1054	2.85	186	2848	25.54	170
1100	2.38	177	2872	31.36	170
1162	2.11	177	3080	35.54	144
1215	2.02	177	3288	42.89	144
1262	2.77	177	3495	51.94	144
1308	3.79	177			
1354	3.66	177			
1400	3.61	177			
1446	3.73	177			

Column I			Column II		
BV	As (μg/l)	C_0	BV	As (μg/l)	C_0
1508	5.33	177			
1562	9.01	171			
1623	9.60	171			
1681	10.47	171			
1738	12.21	171			
1792	12.07	171			
1854	14.75	171			
2186	18.33	202			
2297	19.26	202			
2408	22.35	202			
2518	27.82	202			
2629	36.06	202			
2740	38.16	202			

Column III			Column IV		
BV	As (μg/l)	C_0	BV	As (μg/l)	C_0
24	1.74	88	24	3.05	125
48	0.46	88	56	0.20	125
111	7.18	88	127	2.67	125
159	0.32	88	191	0.37	125
239	0.46	88	255	0.19	125
239	0.92	88	255	5.29	125
318	2.24	88	318	2.73	125
398	9.89	74	446	3.52	149
494	12.79	74	549	5.96	149
605	24.46	74	549	5.83	149
605	18.96	74	637	20.05	149
717	45.31	74	812	20.87	132
828	44.43	105	812	21.01	132
876	48.64	105	876	24.26	132
955	48.40	106	955	28.98	105
1019	49.65	116	1035	53.30	105
1083	52.19	116	1210	52.98	100
1162	65.03	116	1290	37.29	100
1242	65.90	116	1369	34.21	100
1322	63.76	64	1449	47.25	
1401	55.92	64	1529	54.00	84
1529	54.00	100	1529	60.78	84
1608	53.60	88	1608	74.86	84

Column III			Column IV		
BV	As (μg/l)	C_0	BV	As (μg/l)	C_0
1688	45.06	88	1688	53.05	84
1768	38.99	88	1768	38.17	84
1847	43.52	88	1791	57.83	84
1879	47.26	88	1871	61.06	84
1959	56.79	88	1951	70.61	84
2038	64.09	91	2030	88.42	107
2118	52.87	91	2110	101.67	134
2197	51.55	91	2189	120.31	134
2277	61.31	91	2269	130.78	134
2357	73.48	91	2349	123.09	134
2436	79.76	91	2428	97.62	134
2516	99.73	105	2508	95.97	134
2596	96.25	105	2588	82.31	134
2675	102.98	105	2667	78.37	109
2755	77.94	93	2747	101.68	109
2834	79.62	93	2826	107.38	109
2914	84.28	93	2906	80.50	109
2994	81.35	93	2986	69.60	98
3073	72.54	96	3065	55.14	98
3153	91.25	96	3145	78.52	98
3232	94.52	107	3304	85.40	98
3392	95.17	107	3384	90.04	98
3471	105.49	107			

Table S4: Initial and residual concentrations of phosphates, total organic carbon, arsenic, and iron in Pilot A.

Bed Volumes BV	Initial PO_4 mg/l	Res PO_4 mg/l	Initial TOC mg/l	Res TOC mg/l	Initial As μg/l	Res As μg/l	Initial Fe mg/l	Res Fe mg/l
6.3	0.38	0.04	1.38	0.65	122.8	5.97	0.452	0.198
154.1	0.38	0.05	1.41	0.56	107.7	12.13	0.422	0.172
308.4	0.35	0.08	1.79	0.72	106.2	14.44	0.385	0.040
399.4	0.45	0.15	1.54	0.72	109.9	16.65	0.385	0.040
506.6	0.31	0.11	1.47	0.82	105.7	20	0.470	0.026
623.1	0.4	0.2	1.33	1.07	105.3	24.74	0.395	0.025
629.7	0.35	0.22	1.25	0.82	105.2	24.74	0.395	0.025
761.7	0.38	0.25	1.46	0.95	107.1	36.33	0.395	0.025
876.8	0.32	0.22	1.32	0.88	108.4	44.5	0.380	0.090
989.2	0.36	0.28	1.26	0.92	105.2	45.86	0.380	0.090
993.2	0.35	0.3	1.28	1.07	105.1	45.86	0.393	0.121

1001.3	0.37	0.32	1.32	1.11	94.6	44.75	0.473	0.096

Table S5: Initial and residual concentrations of phosphates, total organic carbon, arsenic, and iron in Pilot B.

Bed Volumes BV	Initial PO_4 mg/l	Res PO_4 mg/l	Initial TOC mg/l	Res TOC mg/l	Initial As μg/l	Res As μg/l	Initial Fe mg/l	Res Fe mg/l
5.8	0.34	0.06	1.49	0.36	118.0	0.50	0.433	0.149
9.7	0.30	0.08	1.57	0.27	112.0	0.50	0.391	0.074
95.6	0.23	0.09	1.29	0.30	102.0	0.50	0.383	0.038
185.3	0.41	0.15	1.27	0.22	118.0	3.37	0.386	0.057
268.7	0.31	0.13	1.42	0.31	113.3	4.36	0.416	0.103
453.7	0.45	0.20	1.35	0.42	99.4	8.19	0.366	0.233
740.4	0.32	0.14	1.43	0.31	112.5	23.90	0.433	0.094
932.4	0.39	0.20	1.32	0.45	108.0	36.47	0.362	0.219
1124.4	0.41	0.25	1.32	0.55	113.3	42.97	0.385	0.045
1412.4	0.45	0.31	1.32	0.65	124.3	56.26	0.333	0.054
